# Chirality Transfer and Thiazolidine or Thiazine Formation in Reactions of L and D Enantiomers of β- or γ-Sulfhydryl Amino Acids with Imidazole Carboxaldehydes and Nickel(II)

**DOI:** 10.3390/molecules31132234

**Published:** 2026-06-25

**Authors:** Cynthia T. Brewer, Greg Brewer, Raymond J. Butcher

**Affiliations:** 1 Department of Chemistry, Catholic University, Washington, DC 20064, USA; 2 Department of Chemistry, Howard University, Washington, DC 20059, USA; rbutcher99@yahoo.com

**Keywords:** chirality transfer, thiazolidine, thiazine, cysteine, penicillamine, homocysteine, homochirality

## Abstract

The reaction of either the L or D enantiomer of H_2_N-C*H(R)CO_2_^−^ (R = -CH_2_SH cysteine, C; -C(SH)(CH_3_)_2_, penicillamine, PN; or -CH_2_CH_2_SH, homocysteine, HC) with an imidazole-4-carboxaldehyde and nickel(II) acetate in methanol yields a single stereoisomer of a thiazolidine (from C or PN) or a thiazine (from HC) nickel complex. Five pairs of enantiomeric products were prepared and characterized by IR, ESI MS, EA, and single crystal structure determination. There is retention of chirality for the thiazolidine and thiazine complexes on ring position 4, C_α_ of the parent amino acid, and transfer of chirality to the newly generated stereogenic centers, ring positions 3 (the amino acid nitrogen atom, N_AA_) and 2 (the aldehyde carbon atom, C_ald_). For the thiazolidines, the new stereogenic centers, N_AA_, and C_ald_, have identical stereochemical assignments to one another and to the assignment of the alpha carbon atom, either all R from the L enantiomers of C and PN or all S from the D enantiomers of C and PN. For the thiazine products from HC, the newly generated stereogenic centers, ring positions 3 (N_AA_) and 2 (C_ald_), are identical to one another but opposite to that of the retained stereogenic center (ring position 4, the alpha carbon atom). Regardless of stereochemical assignment (R or S), the hydrogen atoms of C_α_, N_AA_, and C_ald_, ring positions 4, 3, and 2, are always all *cis* to one another for the five pairs of enantiomers examined. This is a consequence of the fact that the thiazolidine and thiazine rings are fused to two other chelate rings of the complexes, which seems to explain the high stereospecificity observed in these systems.

## 1. Introduction

**Figure 1:**
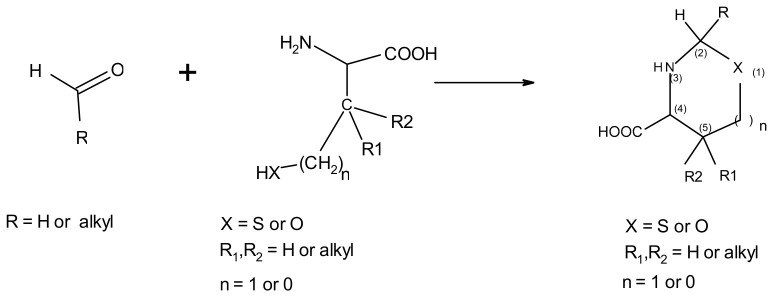
The reaction of formaldehyde (R = H) with a β-hydroxy or sulfhydryl amino acid to give oxazolidines (X = O, n = 0), thiazolidines (X = S, n = 0), or thiazines (X = S, n = 1). The numbering scheme of the rings is shown; O or S is ring position 1 and N is ring position 3. If R is not a H atom, the aldehyde is prochiral.

The reaction of sulfhydryl-containing amino acids with an aldehyde to form thiazolidine rings has been of keen interest because of their potential in areas such as anti-tubercular effects [1], anti-cancer effects [2], neuroprotective effects [3], anti-diabetic effects [4], antioxidant effects [5], and anti-aging [6]. A ^1^H NMR study of the reaction between formaldehyde and various amino acids (AAs) [7] showed that β-hydroxy amino acids (βOHAAs) or β-sulfhydryl amino acids (βSHAAs) gave cyclized products, oxazolidines from a βOHAA such as threonine (T), or thiazolidines from a βSHAA such as cysteine (C). Homocysteine (HC), a ƔSHAA, gave a thiazine (see Figure 1).

The mechanism for the reaction of cysteine with formaldehyde has been examined extensively [8,9]. Several models for thiazolidine formation were examined, and the findings suggested that the formation of thiazolidine occurs by a pathway involving the intermediate formation of a cationic imine (Schiff base), similar to the proposed mechanism for oxazolidine formation from aldehydes and 2-amino alcohols in the presence of transition metals [10].

**Figure 2:**
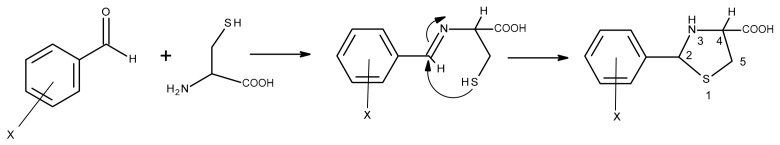
Reaction of substituted benzaldehydes with cysteine.

Reaction of a βSHAA, such as cysteine, with a prochiral aldehyde, such as benzaldehyde and its derivatives, allows for investigation of the stereochemistry [11,12]. Initially an aldimine, -O_2_C-CH(CH_2_SH)-N=CH-Ph, is produced, but subsequent attack of the sulfur nucleophile at the imine carbon atom (former aldehyde carbon atom, C_Ald_) gives thiazolidines with a stereochemistry at ring position 4 identical to that of the former alpha carbon atom, C_α_, of the parent βSHAA (see Figure 2). The stereochemistry at ring position 2 is 50%R and 50%S since attack on C_Ald_ can occur from above or below the imine plane. The resulting product is a mixture of (2R,4R) and (2S,4R) diastereomers. Analysis of ID and 2D NMR data for the product of L-cysteine with o-vanillin resulted in an estimated ratio of 7:8 for the diastereomers, consistent with nearly equal probability of attack on the imine carbon atom from above or below the imine plane [13]. It is possible to separate the diastereomers by recrystallization or column chromatography [14,15]. There is no evidence of chirality transfer in the reactions of a βSHAA with a prochiral aldehyde since the reactions produce a 50:50 mixture of diastereomers. Chirality transfer is the creation of a new chiral center, with a specific stereochemical outcome, in a reaction of a chiral substance with an achiral one [16,17,18].

The reaction of l-cysteine with 3,5-dinitrosalicylaldehyde (another prochiral aldehyde) gives a 50:50 mixture of the diastereomeric thiazolidine-4-carboxylic acid ligands (L^SR^ and L^RR^) [19]. (The first and second superscripts, respectively, are the stereochemical designations of C2 (C_Ald_) and C4 (C_α_) of the thiazolidine.) As before, in the reaction of benzaldehyde with L-cysteine, there is no evidence of chirality transfer since the stereochemistry of the C2 carbon atom formed in the reaction is both S and R. However, reaction of the diastereomers formed from l-cysteine and 3,5-dinitrosalicylaldehyde with LnCl_3_·6H_2_O (Ln = Sm, Eu, or Th) gives only the Λ form of the helical [Ln_2_(L^SR^)_3_(H_2_O)_5_]·3H_2_O complex. The reaction of d-cysteine with 3,5-dinitrosalicylaldehyde and LnCl_3_·6H_2_O gives only the Δ isomer of [Ln_2_(L^RS^)_3_(H_2_O)_5_]·3H_2_O. This work demonstrates chirality transfer in the control of the handedness of a helical assembly (Λ or Δ) by the chirality of the AA used in making the thiazolidine.

Chirality transfer from C_α_ of an amino acid to other second period elements, rather than a stereogenic metal complex, is of greater interest and is also observed. Recently, we reported the syntheses, crystal structures, and chirality transfer in a series of nickel complexes prepared from the reactions of three βOHAAs (both L and D enantiomers) with prochiral nitrogenous aldehydes in the presence of nickel(II) [20]. The resulting eight complexes (four pairs of enantiomers) contained an oxazolidine ring with new stereogenic centers at C2 (former aldehyde carbon atom C_Ald_) and at N_AA_ (the nitrogen atom of the former AA). In each case, the stereochemical assignment of C2 and N_AA_ atoms were opposite to that of the alpha carbon atom. Thus, an L-βOHAA gave an oxazolidine with R assignments for C2 (C_Ald_) and N_AA_ of the oxazolidine while reaction of the D-βOHAA produced the enantiomer. This reaction produces a single stereoisomer of an oxazolidine with stereogenic centers at ring positions 2, 3, and 4.

The purpose of this work is to investigate the reactivity of β- or γSHAAs with three 4-imidazole carboxaldehydes and nickel(II) to determine if thiazolidine or thiazine rings are formed and if chirality transfer from the alpha carbon atom to the former aldehyde carbon atom, C_Ald_, and the AA nitrogen atom, N_AA_, is observed.

## 2. Results and Discussion

### 2.1. Approach, Ligands Employed, and Reactions Examined

**Figure 3:**
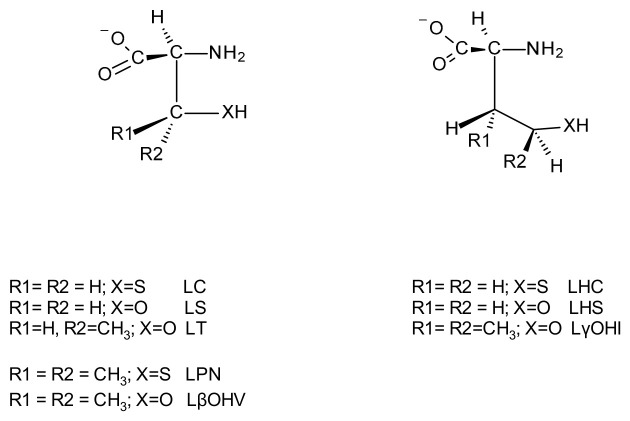
Drawings of the L enantiomers of the three βSHAAs examined (cysteine, C, penicillamine, PN, and homocysteine, HC) and their oxygen analogs (serine, S, threonine, T, valine, V, homoserine, HS, and isoleucine, I).

The aldehydes used were imidazole 4-carboxaldehyde (4Im), 5-methyl-4-imidazole carboxaldehyde (5Me4Im), and 2-methyl-4-imidazole carboxaldehyde (2Me4Im). The β or ƔSHAAs utilized are pictured in Figure 3. To compare and contrast the reactivity of the sulfhydryl and hydroxyl groups, Figure 3 also contains β- or γOHAAs. Reactions of each of the imidazole carboxaldehydes were done with the L enantiomer of the β- or ƔSHAA, and only those reactions that gave crystalline products were repeated with the D enantiomer.

The product in the reaction of a βSHAA with an aldehyde could be an aldimine or it could be a thiazolidine, formed when the imine carbon atom is attacked by the sulfhydryl group of the presumed aldimine intermediate. However, there is only one aldimine of cysteine in the CSD [21], and only thiazolidines are observed here and elsewhere in the literature.

**Figure 4:**
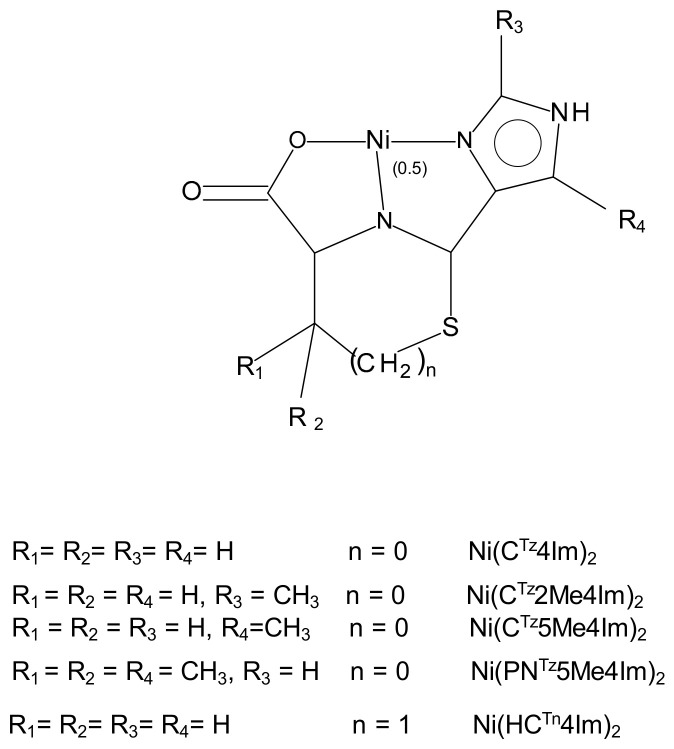
The products for the reactions of the β- or ƔSHAAs with either 4Im, 2Me4Im, or 5Me4Im. The superscripted Tz or Tn indicates a thiazolidine or thiazine ring, respectively.

The colorless reaction mixtures of the β- or ƔSHAA and 4-imidazole carboxaldehydes turned purple upon addition of nickel(II) and over time deposited purple crystals of thiazolidine or thiazine complexes. The products of these reactions are shown in Figure 4. The notation for the ligand is the symbol for the β- or ƔSHAA followed by the symbol for the 4-imidazole carboxaldehyde that was used. A superscript of Tz or Tn is added to indicate whether the product is a thiazolidine or a thiazine.

### 2.2. Preliminary Characterization

FTIR spectra were recorded and are provided in the ESI for the crystalline β- or γSHAA complexes. The complexes show a strong carboxylate band and water bands as they crystallize as hydrates. There was no imine band observed, which is consistent with the XRD data. CHN EA data and ESIMS data were obtained for the complexes derived from the L enantiomer of the β- or γSHAA complexes. The CHN data were in close agreement with the theoretical values. The ESIMS data were useful as a prominent molecular ion; [M + H]^+^, [M + Na]^+^, or [M + K]^+^ were always observed. Several of the complexes also produced a [Ni_2_L_3_]^+^ ion. The crystalline solids obtained from the reaction mixtures were not sufficiently soluble to obtain UV–vis spectra, NMR, or optical rotation data. The ESIMS data and IR spectra are provided in the Electronic Supplemental Information (ESI).

### 2.3. Nickel Coordination Environment

The crystallographic information for the eight thiazolidine complexes (four pairs of enantiomers), two thiazine complexes (one pair of enantiomers), and the one aldimine complex, prepared from a ƔOHAA, is given in Supplementary Table S1. Selected structural parameters for the L enantiomers of the thiazolidine or thiazine complexes are given in Supplementary Table S2 to illustrate the slightly distorted octahedral geometry of the nickel(II) ion. The nickel(II) ion is bound to two identical N_2_O ligands through a carboxylate oxygen atom, O_CA_, the amino acid nitrogen atom, N_AA_, and the imidazole nitrogen atom, N_Im_.

**Figure 5:**
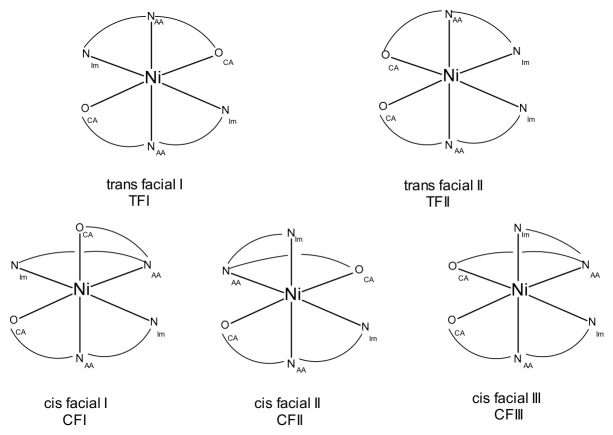
The five modes of *facial* coordination of two unsymmetric tridentate N_2_O ligands around an octahedral metal. The donor atoms in the present complexes are O_CA_, N_AA_, and N_Im_. The top two are named *trans facial (TF)* with *trans* central donor atoms, N_AA_. The bottom three are *cis facial* (CF) with *cis* central donor atoms, N_AA_.

**Figure 6:**
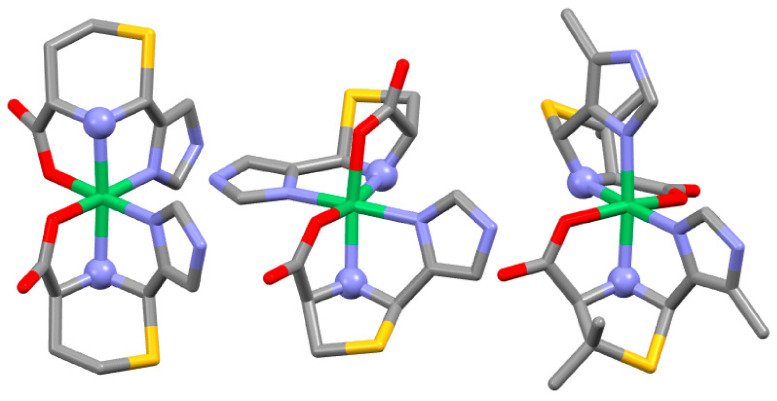
The structures of complexes prepared from the L enantiomers of the β or ƔSHAAs, Ni(HC^Tn^4Im)_2_ (**left**), Ni(C^Tz^4Im)_2_ (**middle**), and Ni(PN^Tz^5Me4Im)_2_ (**right**). For consistency, the ligands in each of the three structures are oriented the same as shown in Figure 5 for each. All hydrogen atoms are omitted for clarity and the central donor atoms, N_AA_, are shown as spheres to allow easy recognition of the mode of coordination. The HC complex exhibits TFII, the C complex is CFI, and the PN complex is CFII.

The mode of coordination of two identical N_2_O ligands prepared by condensation of an AA and a nitrogenous carboxaldehyde to a nickel(II) ion can be *meridional* (blue) or *facial* (purple). The former has been observed in our previously reported aldimine complexes with a hydrocarbon side chain [22], while the latter is found in the present thiazolidine and thiazine complexes, our earlier series of oxazolidine complexes [20] and complexes of PyCH_2_NHCH(R)CO_2_^−^ (R=H or CH_3_) [23], prepared by the borohydride reduction of PyCH=NCH(R)CO_2_^−^. The bis coordination of unsymmetric tridentate ligands (O_CA_N_AA_N_Im_) can result in two *trans facial* geometries with the central N_AA_ atoms *trans* (TFI and TFII) and three *cis facial* geometries with the central N_AA_ atoms *cis* (CFI, CFII and CFIII). This produces five diastereomers, as shown in Figure 5. Each of these has a metal stereogenic center, Δ or Λ enantiomers, except TFI, which is centrosymmetric [24]. The six present thiazolidine complexes of cysteine and the eight previously reported isostructural oxazolidine complexes are all CFI (N_Im_ atoms *trans*), while the two complexes of penicillamine are CFII (O_CA_ atoms *trans*). The HC complexes are TFII (only N_AA_ atoms *trans)*, which allows the bulkier six-membered thiazine rings to be *trans* to one another, reducing steric interactions present in CF modes. There are no prior structures of HC or its complexes in the CSD other than that of HC itself [25]. The TFI (all like atoms *trans*) and CFIII (no like atoms *trans)* geometries are not observed in these systems. Specific examples of TFII, CFI, and CFII are given in Figure 6.

### 2.4. Overall Shape of the Complexes

Each of the five enantiomeric pairs of complexes observed here share an unusual feature in common, a fused three-ring system. For complexes of C or PN, each of the three rings is a five-membered ring. These rings and their component atoms are the carboxylate (Ni, O_CA_, C_CA_, C_α_, and N_AA_), imidazole (Ni, N_Im_, C_Im_, C_ald_, and N_AA_), and thiazolidine (S, C_ald_, N_AA_, C_α_, and C_β_) rings. For the HC complexes, the atoms in the carboxylate and imidazole rings are identical to those given above, and the six-membered thiazine ring atoms are S, C_ald_, N_AA,_ C_α_, C_β_, and Cγ. The bond distances and angles for the L enantiomers of the carboxylate, imidazole, and thiazolidine or thiazine rings are given in Supplementary Table S3. The values are unremarkable given the atoms and hybridizations involved but are provided for completeness.

**Figure 7:**
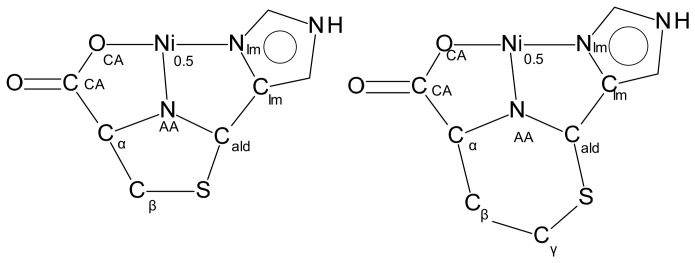
Labeling of the donor atoms of the thiazolidine (**left**) and thiazine (**right**) complexes. The donor atoms, O_CA_, N_AA_, and N_Im_, and the other ligand atoms that define the chelate rings, C_CA_, C_α_, C_β_, C_Ɣ_, S, C_Im_, and C_ald_, are indicated.

The three rings are pictured in Figure 7 for the thiazolidine and thiazine complexes. Each ligand can be pictured as a pseudo three-sided box. The imidazole and carboxylate rings are approximately perpendicular to one another and share an edge, the Ni and N_AA_ atoms. The thiazolidine or thiazine ring is the bottom of the pseudo three-sided box and shares edges, the C_α_ and N_AA_ atoms, and the N_AA_ and C_Ald_ atoms, with the carboxylate and imidazole rings, respectively.

The three fused rings is an unusual feature and has been observed in the products of our earlier reactions of βOHAA with nitrogenous carboxaldehydes [20] and in the nickel(II) and cobalt(III) complexes of (2R,4R)-2-(2-pyridyl)-thiazolidine-4-carboxylic acid [26].

### 2.5. Chirality Transfer

**Figure 8:**
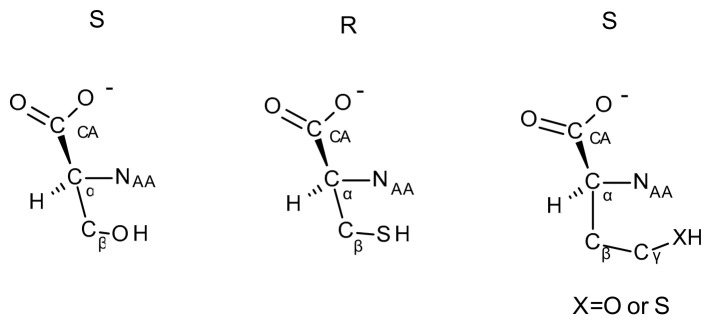
The L enantiomers of a βOHAA (**left**), a βSHAA (**middle**), and a ƔXHAA (**right**). All share the same orientation about C_α_, but the βOHAA (**left**) and the βSHAA (**middle**) differ in designation (S for a βOHAA (**left**) and R for a βSHAA (**middle**)) due to man-made priority rules. For a ƔOH or ƔSH AA (**right**), there is no difference due to the distance of the O or S atoms from C_α_; both are S. The assignment of C_α_ as S or R depends in part on the substituent on C_β_, an oxygen or a sulfur atom. For the βOHAA, C_β_ is the 3rd priority off C_α_, while for the βSHAA, C_β_ is the 2nd priority off C_α_. This man-made distinction reverses the designation of C_α_ between the βOHAA and the βSHAA, even though there is no difference in the orientation of groups, as illustrated.

The most important aspect of this work is that the stereochemistry of the two newly generated stereogenic centers, N_AA_ and C_ald_, is determined by the chirality of C_α_. Table 1 gives the designation for the metal stereogenic center and those of C_α_, N_AA_, and C_Ald_ for the five enantiomeric pairs of complexes prepared from C (three pairs), PN (one pair), and HC (one pair). Before examining the results, it is important to notice differences in the designation (R or S) of the L enantiomers of βOHAAs, βSHAAs, and ƔSHAAs pictured in Figure 8. It is important to keep this difference in mind when comparing chirality transfer results for oxazolidines, prepared from βOHAAs, and thiazolidines, prepared from βSHAAs (C and PN).

The observed chirality transfer in the oxazolidines, prepared from βOHAAs [20], was that an S C_α_ gave a Λ complex with an R N_AA_ and an R C_ald_. Conversely, use of an R C_α_ gave a Δ complex with an S N_AA_ and an S C_ald_. The chirality transfer for the six complexes of both L and D enantiomers of cysteine with 4Im, 5Me4Im, and 2Me4Im should be identical to that of the oxazolidines, with the understanding that an S βOHAA corresponds to an R βSHAA, as all exhibit CFI coordination. Inspection of Table 1 shows that for the six cysteine complexes, this prediction is verified.

**Figure 9:**
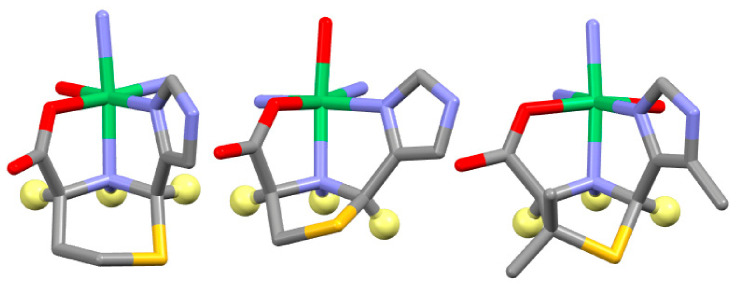
The structures of the L enantiomers of Ni(HC^Tn^4Im)_2_ (**left**), Ni(C^Tz^4Im)_2_ (**middle**), and Ni(PN^Tz^5Me4Im)_2_ (**right**), as shown in Figure 6. For clarity, the upper ligand, except for its donor atoms, and all hydrogen atoms, other than those bound to C_α_, N_AA_, and C_Ald_ (shown as yellow spheres), have been deleted. Note that these three hydrogen atoms are always *cis* to one another.

For the oxazolidines, thiazolidines, and thiazines, the key stereochemical requirement is that the three hydrogen atoms of C_α_, N_AA_, and C_Ald_ must be *cis* to one another as pictured in Figure 9. Inversion of any one of the hydrogen atoms would require movement of its bonded partner. Energetically, this would be very difficult as all are bonded to atoms that are part of the three fused rings.

The chirality transfer of the PN complexes is that an R C_α_ gives a Δ complex and R assignments for N_AA_ and C_Ald_ and that an S C_α_ gives a Λ complex and S assignments for N_AA_ and C_Ald_. The difference between the C and PN complexes is that the former are CFI and the latter are CFII. CFI and CFII are diastereomers of one another. Since the mode of coordination of the C and PN ligands is different, we cannot expect an identical chirality transfer to the metal complex. The correlation of the metal stereochemistry, Δ or Λ, with that of C_α_ is due to energy differences between the diastereomeric ΔC_α_ and ΛC_α_ complexes [27,28,29]. The three hydrogen atoms of C_α_, N_AA_, and C_Ald_ in the PN complexes are *cis* to one another. For the βSHAA complexes, this fact requires that the assignments of these three atoms are RRR or SSS. This is observed for all the C and PN complexes.

The observed chirality transfer for the HC complexes, which are TFII, is that L-homocysteine, with an S C_α_ (see Figure 8), gives a Λ complex and R assignments for N_AA_ and C_ald._ For the enantiomer, the reverse outcome is observed.

**Figure 10:**
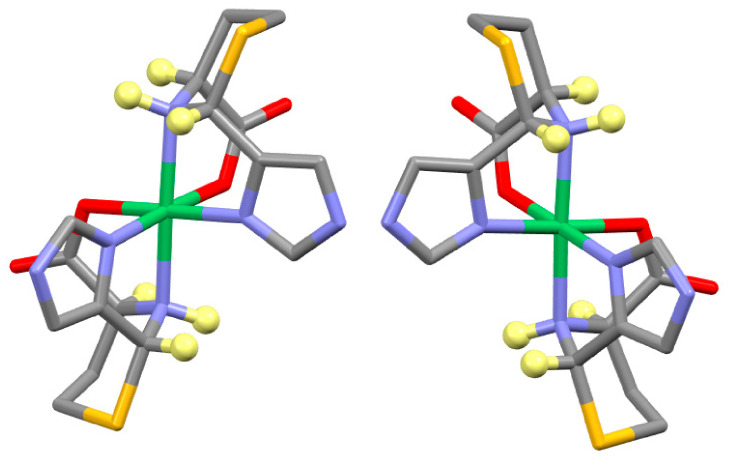
The L (**left**) and D (**right**) enantiomers of Ni(HC^Tn^4Im)_2_. For clarity, all hydrogen atoms except those bound to C_α_, N_AA_, and C_Ald_ (shown as yellow spheres) have been deleted. Note the importance of the *cis* arrangement of these three hydrogen atoms. The bulkier six-membered thiazine ring (chair conformation) exhibits a TFII mode of coordination.

The stereochemical observation for the previously reported eight oxazolidine and the present eight thiazolidine and two thiazine complexes is that the hydrogen atoms of C_α_, N_AA_, and C_Ald_ are always *cis* to one another. The designation of the chirality of C_α_, N_AA_, and C_Ald_ (RRR, SRR, or any other assignment) is a consequence of the *cis* stereochemistry of the product. It is not energetically possible for one of these hydrogen atoms to be *trans* to the other two as this would require inversion of its bonded partner (C_α_, N_AA_, or C_Ald_), which is an atom in the three fused rings. This is illustrated in Figure 10 for the enantiomers of the HC complex.

These ten thiazolidine/thiazine complexes provided here and the earlier nine complexes of oxazolidines [20] provide significant examples of chirality transfer. The stereochemical assignments of the two newly generated chiral centers, N_AA_ and C_Ald_, depend only on C_α_ of the amino acid. l-cysteine only gives a thiazolidine complex with the three hydrogen atoms of the C_α_^R^N_AA_^R^C_Ald_^R^ triad *cis*. This result does not suggest that the other three diastereomers, C_α_^R^N_AA_^R^C_Ald_^S^, C_α_^R^N_AA_^S^C_Ald_^R^, and C_α_^R^N_AA_^S^C_Ald_^S^, are not formed, but they are not isolated. There are two possibilities: either all four diastereomers are formed (at 25% for each), but only one reacts with nickel(II) or there is preference for the formation of the C_α_^R^N_AA_^R^C_Ald_^R^ diastereomer. Future solution studies of the reaction mixture, not the product, are needed to investigate this. The isolated reaction product is the C_α_^R^N_AA_^R^C_Ald_^R^ complex in greater than 50% yield. This result cannot be explained by statistics, but equilibrium may offer a clue. The reaction of L-cysteine with 4Im likely generates all four of the thiazolidine diastereomers, but only the C_α_^R^N_AA_^R^C_Ald_^R^ diastereomer, with all hydrogen atoms *cis*, allows the three donor atoms O_CA_, N_AA_, and N_Im_ to bind in a *facial* manner to the metal and precipitate out. The orientation of the donor atoms to bind to the metal is a consequence of the stereochemistry of the C_α_^R^N_AA_^R^C_Ald_^R^ triad. The other three diastereomers do not have the three donor atoms oriented to bind in this manner. They stay in solution and can re-equilibrate at each position except C_α_ since a thiazolidine is in equilibrium with l-cysteine and 4Im and the rate of racemization of the non-metal bound nitrogen atom is fast. Repetition of this process over time would result in a significant conversion to a single enantiomer, as is observed here.

### 2.6. Reactivity Comparisons

It is possible to do a qualitative nucleophilic reactivity comparison between the sulfhydryl and hydroxyl groups by comparing the present results using βSHAAs with earlier data using βOHAAs. The likely initial product of the reaction of an aldehyde with either βSHAAs or βOHAAs is the aldimine. If the substituent, SH or OH, is sufficiently nucleophilic, the initial product will cyclize to give a thiazolidine or an oxazolidine, respectively. It is clear that the sulfhydryl group is significantly more reactive than the hydroxyl group in attacking the imine carbon atom. This is illustrated by comparing the products of S (a primary alcohol) and C (a primary thiol) with a 4-imidazole carboxaldehyde in the presence of nickel(II). The former yields only aldimine while the latter yields the cyclized thiazolidine. T, βOHV, and βOHL react with 4-imidazole carboxaldehydes in the presence of nickel(II) to give oxazolidines. These are secondary and tertiary alcohols and would be expected to be more nucleophilic than the primary alcohol S. PN (a tertiary thiol) gives a thiazolidine as anticipated since C (a primary thiol) also gives a thiazolidine. This reactivity comparison can be extended to the ƔOHAA, Ɣ-hydroxyisoleucine (ƔOHI). ƔOHI (a secondary alcohol) gives the structurally characterized aldimine product reported here. In contrast, homocysteine (HC) (a primary thiol and a ƔSHAA) gives the cyclized thiazine product. These observations are consistent with general trends regarding the greater nucleophilicity of sulfhydryl over hydroxyl groups.

## 3. Experimental

### 3.1. General

d-cysteine, d- and l-homocysteine, l-penicillamine, 4-hydroxy-l-isoleucine, 4-imidazolecarboxaldehyde, and 5-methyl-4-imidazolecarboxaldehyde were obtained from Combi-Blocks (San Diego, CA, USA). l-cysteine, nickel(II) acetate tetrahydrate, and methanol were obtained from Aldrich (St. Louis, MO, USA). D-penicillamine was obtained from AAblocks (San Diego, CA, USA.) 2-Methyl-4-imidazolecarboxaldehyde was obtained from ACHEMBLOCK (Hayward, CA, USA). A solution of 1.0 M KOH in methanol was obtained from Supelco (Bellefonte, PA, USA). All solvents were of reagent grade and were used without further purification. IR spectra were obtained on a Perkin Elmer Spectrum Two FT IR spectrophotometer.

### 3.2. External Laboratories

ESI-MS were provided by Axis Pharm Laboratory (San Diego, CA, USA). EA data were provided by Galbraith Laboratories, Inc. (Knoxville, TN, USA).

### 3.3. X-Ray Crystallography

Crystal data for all complexes were collected on a Rigaku Synergy-S single-crystal X-ray diffractometer. All structures were solved using the direct methods program SHELXS-97 [30]. All nonsolvent heavy atoms were located using subsequent difference Fourier syntheses. The structures were refined against F 2 with the program SHELXL [31,32], in which all data collected were used including negative intensities. All nonsolvent heavy atoms were refined anisotropically. All hydrogen atoms were located by Fourier difference. Selected crystallographic details for all complexes are given in Supplementary Tables S1–S3 in the ESI.

### 3.4. Syntheses

Syntheses of the compounds in this study followed the general procedure below for the reaction of d-cysteine with 4-imidazolecarboxaldehyde. d-cysteine (0.121 g, 1.0 mmol) and 4-imidazolecarboxaldehyde (0.096 g, 1.0 mmol) were added to a 100 mL round bottom flask containing 5 mL of water, 10 mL of a solution that was 0.1 M KOH in methanol, and 15 mL of methanol. The reaction mixture was refluxed for 30–40 min. It was removed from reflux, and 10 mL of a solution that was 0.05 M Ni(acetate)_2_·4H_2_O in methanol was added to the still-warm solution. After several days, crystals formed, which were suitable for X-ray analysis.

Supplementary Table S4 of the ESI contains yield, elemental analysis data, and electron spray ionization mass spectral data for a single enantiomer of each of the complexes reported in this study.

## 4. Conclusions

Reaction of cysteine with three different 4-imidazole carboxaldehydes and nickel(II) generates NiL_2_ complexes where L is a tridentate thiazolidine that binds through a carboxylate oxygen atom, O_CA_, the amino acid, N_AA_, and imidazole, N_Im_, nitrogen atoms in a *facial* manner. New chiral centers are generated at N_AA_ and C_ald_, and their assignment is the same as that of the alpha carbon atom, C_α_, of the amino acid. Thus, the new stereogenic centers, C2 (C_Ald_) and N_AA_, are RR if l-cysteine is used and SS if d-cysteine is used. The L and D enantiomers of penicillamine react as does cysteine, giving the same stereochemical outcome. The reaction with HC produces the six-membered thiazine ring where the stereochemical assignments of C_Ald_ and N_AA_ are the reverse of C_α_. The present work with thiazolidines and thiazines and our earlier work with oxazolidines provides nineteen examples with one observed stereochemical conclusion regarding the Ni(II) complexes. The hydrogen atoms of C_α_N_AA_C_Ald_ (ring positions 2, 3, and 4) must be *cis*, regardless of stereochemical assignment. The reason for the crucial *cis* requirement of these three hydrogen atoms is that a *cis* arrangement allows the three donor atoms, O_CA,_ N_AA_, and N_Im_, to bind in a *facial* manner. These reactions demonstrate significant chirality transfer and offer a synthetic route to chiral thiazolidines and thiazines with fixed chirality at ring positions 2, 3, and 4.

## Figures and Tables

**Table 1:** Stereochemistry of the complexes.

Compound	Complex	C_α_	N_AA_	C_Ald_
Ni(LC^Tz^4Im)_2_	Ʌ	R	R	R
Ni(DC^Tz^4Im)_2_	Δ	S	S	S
Ni(LC^Tz^5Me4Im)_2_	Ʌ	R	R	R
Ni(DC^Tz^5Me4Im)_2_	Δ	S	S	S
Ni(LC^Tz^2Me4Im)_2_	Ʌ	R	R	R
Ni(DC^Tz^2Me4Im)_2_	Δ	S	S	S
Ni(LPN^Tz^5Me4Im)_2_	Δ	R	R	R
Ni(DPN^Tz^5Me4Im)_2_	Λ	S	S	S
Ni(LHC^Tn^4Im)_2_	Λ	S	R	R
Ni(DHC^Tn^4Im)_2_	Δ	R	S	S

## Data Availability

All of the cif files for the reported complexes are deposited with the CSD of the CCDC, and the deposition numbers of all complexes are given in Supplementary Table S1. The author will respond to any reasonable request to supply any additional information.

## Supplementary Materials

The following supporting information can be downloaded at: https://www.mdpi.com/article/10.3390/molecules31132234/s1. The ESI contains tables for crystallographic data, bond dis-tances and angles for the coordination sphere, bond distances and angles for the carboxylate, im-idazole/pyridine and oxazolidine rings, CHN analysis data, FTIR of all complexes, and ESIMS spectra.
