# Supplementary material for: Chirality Transfer and Thiazolidine or Thiazine Formation in Reactions of L and D Enantiomers of β- or γ-Sulfhydryl Amino Acids with Imidazole Carboxaldehydes and Nickel(II)
Source: Molecules. 2026 Jun 25;31(13):2234. doi: 10.3390/molecules31132234 (PMC13363088; doi:10.3390/molecules31132234)
Supplement: Supplementary file 1 [file molecules-31-02234-s001.zip › molecules-4375520-supplementary.pdf]

Electronic Supplemental Information for

Chirality Transfer and Thiazolidine or Thiazine Formation in Reactions of L and D enantiomers of β or γ Sulfhydryl Amino Acids with 4-Imidazole Carboxaldehydes and Nickel(II).

Supplemental Table S1 Crystallographic Data for eleven Nickel complexes of β and ɣ Sulfhydryl AA condensed with 4-imidazole carboxaldehydes

Supplemental Table S2. Selected nickel(II) bond distances (Å) and angles (°) of the L β and ɣ Sulfhydryl amino acids condensed with 4-imidazole carboxaldehydes

Supplemental Table S3. Selected bond distances(Å) and angles(°) for the three fused rings of the of the nickel(II) complexes of the L β and ɣ Sulfhydryl amino acids condensed with 4-imidazole carboxaldehydes

Supplemental Table S4. Summary of %yield, EA and ESI MS values for one enantiomer of the prepared complexes

Molecules-Chemical Compounds Characterization Checklist

ESI MS for a single enantiomer of the complexes

FTIR for L and D enantiomers of the β and ɣ Sulfhydryl amino acids condensed with 4-imidazole carboxaldehydes

Supplemental Table S1 Crystallographic Data for all Complexes

Compound	**Ni(LC^{Tz}4Im)$_2$**	**Ni(DC^{Tz}4Im)$_2$**	**Ni(LC^{Tz}2Me4Im)$_2$**	**Ni(DC^{Tz}2Me4Im)$_2$**
CSD number	2427242	2488357	2427221	2488361
Empirical formula	$C_{14}H_{16}N_6NiO_4S_2$ ·$3H_2O$	$C_{14}H_{16}N_6NiO_4S_2$ ·$3H_2O$	$C_{16}H_{20}N_6NiO_4S_2$ ·H_2O	$C_{16}H_{20}N_6NiO_4S_2$ ·H_2O
M/ g mol^{-1}	509.20	509.20	501.22	501.22
Temperature /K	100(2)	100(2)	100(2)	100(2)
λ/ Å	1.54184	1.54184	1.54184	1.54184
Crystal System	Orthorhombic	Orthorhombic	Tetragonal	Tetragonal
Space group	$P2_12_12_1$	$P2_12_12_1$	$P4_32_12$	$P4_12_12$
Unit cell dimensions	a=9.16241(5) Å, b=9.72953(4) Å, c=22.42096(9) Å, α= 90° β=90° γ= 90°	a=9.18380(10) Å, b=9.76610(10) Å, c=22.4044(2) Å, α= 90° β=90° γ= 90°	a=9.25530(2) Å, b=9.25530(2) Å, c=22.27768(9) Å, α= 90° β=90° γ= 90°	a=9.25601(3) Å, b=9.25601(3) Å, c=22.27357(13) Å, α= 90° β=90° γ= 90°
Volume/ $Å^3$	1998.739(15)	2009.45(3)	1998.321(11)	1908.259(17)
Z	4	4	4	4
Abs. Coeff./mm^{-1}	3.830	3.810	3.922	3.922
F(000)	1056	1056	1040	1040
Crystal size/ mm^3	0.427x0.115x0.042	0.374x0.191x0.074	0.220x0.179x0.097	0.496x0.392x0.258
Theta range/°	3.943 to 76.114	3.946 to 76.333	5.175 to 76.299	5.175 to 76.429
Index ranges	$-11\leq h\leq 9$ $-12\leq k\leq 12$ $-28\leq l\leq 27$	$-11\leq h\leq 11$ $-12\leq k\leq 11$ $-26\leq l\leq 28$	$-11\leq h\leq 11$ $-11\leq k\leq 11$ $-27\leq l\leq 27$	$-11\leq h\leq 11$ $-11\leq k\leq 11$ $-28\leq l\leq 22$
Reflections Collected	47754	35938	66864	36509
Independent Reflections	4114	4169	1991	2000
Flack param	-0.007(4)	0.005(10)	-0.008(4)	-0.010(5)
R1	0.0219	0.0393	0.0176	0.0214
wR2	0.0568	0.1029	0.0457	0.0588
GOF on F^2	1.055	1.051	1.076	1.110

Supplemental Table S1 cont.

Compound	**Ni(LC^{Tz}5Me4Im)$_2$**	**Ni(DC^{Tz}5Me4Im)$_2$**	**Ni(LPN^{Tz}5Me4Im)$_2$**	**Ni(DPN^{Tz}5Me4Im)$_2$**
CSD number	2496227	2488359	2427220	2488358
Empirical formula	$C_{16}H_{20}N_6NiO_4S_2$	$C_{16}H_{20}N_6NiO_4S_2$	$C_{20}H_{28}N_6NiO_4S_2$ $\cdot CH_3OH \cdot 2\ H_2O$	$C_{20}H_{28}N_6NiO_4S_2 \cdot 3\ H_2O$
M/ g mol^{-1}	483.21	483.21	607.39	593.36
Temperature /K	100(2)	100(2)	100(2)	100(2)
λ/ Å	1.54184	1.54184	1.54184	1.54184
Crystal System	Monoclinic	Monoclinic	Monoclinic	Monoclinic
Space group	I2	I2	$P2_1$	$P2_1$
Unit cell dimensions	a=9.0511(2) Å,<br>b=5.5328(3) Å,<br>c=19.3778(6) Å,<br>α= 90°<br>β=94.108(3)°<br>γ= 90°	a=9.03841(10) Å,<br>b=5.2984(5)) Å,<br>c=19.36598(17) Å,<br>α= 90°<br>β=94.3361(9)°<br>γ= 90°	a=12.13180(10) Å,<br>b=10.35780(10) Å,<br>c=12.44600(10) Å,<br>α= 90°<br>β=118.08060(10)°<br>γ= 90°	a=12.0615(2) Å,<br>b=10.40070(10) Å,<br>c=12.4282(2) Å,<br>α= 90°<br>β=117.742(2)°<br>γ= 90°
Volume/ Å^3	967.91(6)	965.159(16)	1379.78(2)	1379.88(4)
Z	2	2	2	2
Abs. Coeff./mm^{-1}	3.803	3.814	2.867	2.853
F(000)	500	500	640	624
Crystal size/ mm^3	0.116x0.072x0.032	0.108x0.071x0.032	0.382x0.308x0.112	0.254x0.161x0.020
Theta range/°	4.575 to 75.977	4.580 to 76.140	4.026 to 76.089	4.019 to 76.417
Index ranges	$-11\leq h\leq 11$<br>$-6\leq k\leq 5$<br>$-24\leq l\leq 24$	$-9\leq h\leq 11$<br>$-6\leq k\leq 6$<br>$-24\leq l\leq 24$	$-15\leq h\leq 13$<br>$-12\leq k\leq 9$<br>$-15\leq l\leq 15$	$-14\leq h\leq 15$<br>$-13\leq k\leq 12$<br>$-15\leq l\leq 15$
Reflections Collected	14762	16106	24145	50561
Independent Reflections	1825	1896	5074	5586
Flack param	0.01(2)	-0.012(14)	-0.012(6)	0.001(8)
R1	0.0510	0.0350	0.0252	0.0355
wR2	0.1343	0.0904	0.0637	0.0955
GOF on F^2	1.109	1.022	1.036	1.062

Supplemental Table S1 cont.

Compound	**Ni(LHC^{Tn}4Im)$_2$**	**Ni(DHC^{Tn}4Im)$_2$**	**Ni(βOHI^{Ald}5Me4Im)$_2$**
CSD number	2427234	2496228	2427235
Empirical formula	$C_{16}H_{20}N_6NiO_4S_2$ 5 H_2O	$C_{16}H_{20}N_6NiO_4S_2$ 5 H_2O	$C_{22}H_{32}N_6NiO_6$ ·4H_2O
M/ g mol^{-1}	573.29	573.29	607.31
Temperature /K	100(2)	100(2)	100(2)
λ/ Å	1.54178	1.54178	1.54184
Crystal System	Monoclinic	Monoclinic	Orthorhombic
Space group	$P2_1$	$P2_1$	$P2_12_12_1$
Unit cell dimensions	a=8.55494(6) Å, b=14.69195(9) Å, c=9.28138(5) Å, α= 90° β=91.1261(6)° γ= 90°	a=8.55970(10) Å, b=14.7117(2) Å, c=9.29520(10) Å, α= 90° β=91.8760(10)° γ= 90°	a=9.18325(4) Å, b=15.25269(6) Å, c=20.43168(8) Å, α= 90° β=90° γ= 90°
Volume/ Å^3	1165.905(12)	1169.90(2)	2861.85(2)
Z	2	2	4
Abs. Coeff./mm^{-1}	3.419	3.407	1.515
F(000)	600	600	1056
Crystal size/ mm^3	0.119x0.072x0.053	0.209x0.123x0.058	0.331x0.181x0.127
Theta range/°	4.767 to 76.290	4.760 to 76.005	3.616 to 76.311
Index ranges	$-10\leq h\leq 9$ $-18\leq k\leq 18$ $-11\leq l\leq 11$	$-10\leq h\leq 10$ $-15\leq k\leq 18$ $-11\leq l\leq 11$	$-11\leq h\leq 8$ $-19\leq k\leq 19$ $-25\leq l\leq 25$
Reflections Collected	40491	94739	77555
Independent Reflections	4725	4552	5867
Flack param	-0.011(4)	0.002(9)	-0.026(5)
R1	0.0198	0.0419	0.0247
wR2	0.0542	0.1119	0.0665
GOF on F^2	1.052	1.070	1.038

Supplemental Table S2. Selected nickel(II) bond distances (Å) and angles (°)

a) for the thiazolidine (Tz) complexes of LC with N_{Im}-Ni-N'$_{Imi}$ trans. Values for the D enantiomers, available from the CCDC, do not differ significantly. An atom marked with a ' indicates that it is an atom on the second of two identical ligands. There is only one value for each entry of a complex if the asymmetric unit contains only one of the two ligands.

Complex/ parameter	Ni(LC^{Tz}4Im)$_2$	Ni(LC^{Tz}2Me4Im)$_2$	Ni(LC^{Tz}5Me4Im)$_2$
Ni-O_{CA}	2.062(16)<br>2.0739(17))	2.0707(12)	2.070(5)
Ni-N_{AA}	2.132(2)<br>2.1518(19)	2.1254(16)	2.142(6)
Ni-N_{Im}	2.073(2)<br>2.0443(19)	2.0552(15))	2.060(4)
$N_{Im/}$-Ni-N'$_{Im}$	173.90(8)	176.90(9)	177.9(4)
O_{CA}-Ni-N'$_{AA}$	168.72(7)<br>169.37(8)	177.03(6)	164.92(16)
O_{CA}-Ni-N_{Im}	93.20(7)<br>92.74(7)	86.77(6)	95.9(2)

Supplemental Table S2. Selected nickel(II) bond distances (Å) and angles (°)

b) for the thiazolidine (Tz) complex of PN with O_{CA}-Ni-O'_{CA} trans and

c) thiazine (Tzn) complex of LHC with N_{AA}-Ni-N_{AA} trans,.

Values for the enantiomer, available from the CCDC, do not $differ_{AA}$ significantly. An atom marked with a ' indicates that it is an atom on the second of two identical ligandsThere is only one value for each entry of a complex if the asymmetric unit contains only one of the two ligands.

.

Complex/ Parameter	$Ni(LPN^{Tz}5Me4Im)_2$		Complex/ parameter	$Ni(LHC^{Tzn}4Im)_2$
Ni-O_{CA}	2.050(2)<br>2.081(2)		Ni-O_{CA}	2.0742(15)<br>2.0408(15)
Ni-N_{AA}	2.122(2)<br>2.106(2)		Ni-N_{AA}	2.1362(18)<br>2.1531(18)
Ni-N_{Im}	2.020(2)<br>2.044(2)		Ni-N_{Im}	2.0498(18)<br>2.0421(18)
O_{CA}-Ni-O'_{CA}	166.80(8)		N_{AA}-Ni-N'_{AA}	I75.68(7)
N_{AA}-Ni-$N'_{Im/}$	175.86(9)<br>172.33(11)		O_{CA}-Ni-N'_{Im}	179.01(7)<br>176.83(7)
O_{CA}-Ni-N_{Im}	92.43(10)<br>97.72(9)		O_{CA}-Ni-N_{Im}	86.92(7)<br>86.60(7)

Supplemental Table S3. Selected bond distances(Å) and angles(°) for the three five membered rings of the L complexes of C (a, b and c) and PN (d) and for the two five membered rings and one six membered rings of L HC (e). Values for the D enantiomers are available from the CCDC and do not significantly differ from the L enantiomer

a) Ni(LC^{Tz}4Im)$_2$ Imidazole: (Ni N_{Im} C_{Im} C_{ald} N_{AA}), Carboxylate: (Ni O_{CA} C_{CA} C_α N_{AA}) and Thiazolidine: (N_{AA} C_α C_β S_{Tz} C_{ald}) There are two values for each entry if there are two ligands in the asymmetric unit.

Imidazole	Value	Carboxylate	value	Thiazolidine	value
Ni- N_{Im}	2.073(2)<br>2.0443(19)	Ni -O_{CA}	2.0262(16)<br>2.0739(17)	N_{AA}-C_α	1.488(3)<br>1.481(3)
N_{Im}-C_{Im}	1.373(3)<br>1.376(3)	O_{CA}-C_{CA}	1.262(3)<br>1.259(3)	C_α-C_β	1.523(3)<br>1.524(3)
C_{Im}-C_{ald}	1.498(3)<br>1.497(3)	C_{CA}-C_α	1.545(3)<br>1.534(3)	C_β -S	1.807(3)<br>1.814(3)
C_{ald}-N_{AA}	1.494(3)<br>1.495(3)	C_α-N_{AA}	1.488(3)<br>1.481(3)	S-C_{ald}	1.837(2)<br>1.837(2)
N_{AA}-Ni	2.132(2)<br>2.1518(19)	N_{AA}-Ni	2.132(2)<br>2.1518(10)	C_{ad} -N_{AA}	1.494(3)<br>1.495(3)
Ni- N_{Im} -C_{Im}	114.60(15)<br>115.65(14)	Ni -O_{CA}-C_{CA}	116.60(14)<br>115.70(15)	N_{AA}-C_α-C_β	108.21(19)<br>107.84(19)
N_{Im}-C_{Im}-C_{ald}	119.8(2)<br>119.5(2)	O_{CA}-C_{CA}-C_α	117.46(19)<br>117.9(2)	C_α -C_β -S	102.45(15)<br>102.45(15)
C_{Im}-C_{ald} -N_{AA}	111.77(19)<br>111.23(18)	C_{CA}-C_α-N_{AA}	109.89(18)<br>110.82(18)	C_β-S-C_{ald}	90.68(11)<br>90.80(12)
C_{ald}-N_{AA}-Ni	112.52(14)<br>112.36(13)	C_α-N_{AA}-Ni	106.25(14)<br>105.95(13)	S-C_{ald} -N_{AA}	107.90(15)<br>108.16(16)
N_{AA}-Ni—N_{Im}	81.34(8)<br>80.97(8)	N_{AA}-Ni-O_{CA}	80.05(7)<br>79.03(7)	C_{ald}-N_{AA}-C_α	110.43(18)<br>110.33(19)

Supplemental Table S3 continued.

b) Ni(LC^{Tz}2Me4ImIm)$_2$. Imidazole: (Ni N_{Im} C_{Im} C_{ald} N_{AA}), Carboxylate: (Ni O_{CA} C_{CA} C_α N_{AA}) and Thiaazolidine: (N_{AA} C_α C_β S C_{ald}). There is only one value for each entry if there is only a single ligand in the asymmetric unit.

Imidazole	Value	carboxylate	Value	thiazolidine	value
Ni-N_{Im}	2.0552(15)	Ni -O_{CA}	2.0707(12)	N_{AA}-C_α	1.485(2)
N_{Im} -C_{Im}	1.383(2)	O_{CA}-C_{CA}	1.265(2)	C_α-C_β	1.522(2)
C_{Im} -C_{ald}	1.503(3)	C_{CA}-C_α	1.540(3)	C_β -S	1.8129(19)
C_{ald}-N_{AA}	1.488(2)	C_α-N_{AA}	1.485(2)	S-C_{ald}	1.8526(18)
N_{AA}-Ni	2.1254(16)	N_{AA}-Ni	2.1254(16)	C_{ald} -N_{AA}	1.488(2)
Ni- N_{Im} -C_{Im}	114.25(12)	Ni-O_{CA}-C_{CA}	115.91(11)	N_{AA}-C_α-C_β	107.88(14)
N_{Im} -C_{Im}-C_{ald}	119.25(16)	O_{CA}-C_{CA}-C_α	116.44(15)	C_α -C_β -S	103.61(12)
C_{Im}-C_{ald}-N_{AA}	111.23(15)	C_{CA}-C_α-N_{AA}	110.99(14)	C_β-S-C_{ald}	92.48(8)
C_{ald}-N_{AA}-Ni	112.29(11)	C_α-N_{AA}-Ni	105.34(11)	S-C_{ald} -N_{AA}	107.64(11)
N_{AA}-Ni-N_{Im}	81.80(6)	N_{AA}-Ni-O_{CA}	80.39(5)	C_{ald}-N_{AA}-C_α	110.52(14)

Supplemental Table S3 continued.

c) Ni(LC^{Tz}5Me4ImIm)$_2$. Imidazole: (Ni N_{Im} C_{Im} C_{ald} N_{AA}), Carboxylate: (Ni O_{CA} C_{CA} C_α N_{AA}) and Thiazolidine: (N_{AA} C_α C_β S C_{ald}). There is only one value for each entry if there is only a single ligand in the asymmetric unit.

Imidazole	Value	carboxylate	Value	thiazolidine	Value
Ni-N_{Im}	2.060(4)	Ni -O_{CA}	2.070(5)	N_{AA}-C_α	1.507(8)
N_{Im} -C_{Im}	1.353(9)	O_{CA}-C_{CA}	1.279(8)	C_α-C_β	1.540(9)
C_{Im} -C_{ald}	1.509(9)	C_{CA}-C_α	1.528(11)	C_β -S	1.795(8)
C_{ald}-N_{AA}	1.485(8)	C_α-N_{AA}	1.507(8)	S_{Tz}-C_{ald}	1.822(6)
N_{AA}-Ni	2.142(6)	N_{AA}-Ni	2.142(6)	C_{ald} -N_{AA}	1.485(8)
Ni- N_{Im} -C_{Im}	115.3(4)	Ni-O_{CA}-C_{CA}	113.1(5)	N_{AA}-C_α-C_β	107.6(5)
N_{Im} -C_{Im}-C_{ald}	119.5(5)	O_{CA}-C_{CA}-C_α	118.1(6)	C_α -C_β -S	105.4(5)
C_{Im}-C_{ald}-N_{AA}	109.3(6)	C_{CA}-C_α-N_{AA}	112.2(5)	C_β-S-C_{ald}	88.0(3)
C_{ald}-N_{AA}-Ni	112.1(4)	C_α-N_{AA}-Ni	108.2(4)	S-C_{ald} -N_{AA}	107.7(4)
N_{AA}-Ni—N_{Im}	79.5(2))	N_{AA}-Ni-O_{CA}	81.9(2)	C_{ald}-N_{AA}-C_α	110.8(5)

Supplemental Table S3 continued

d) $Ni(LPN^{Tz}5Me4Im)_2$. Imidazole: (Ni N_{Im} C_{Im} C_{ald} N_{AA}), Carboxylate: (Ni O_{CA} C_{CA} C_{α} N_{AA}) and Thiazolidine: (N_{AA} C_{α} C_{β} S C_{ald}) There are two values for each entry if there are two ligands in the asymmetric unit.

Imidazole	Value	Carboxylate	Value	Thiazolidine	value
Ni- N_{Im}	2.020(2)<br>2.044(2)	Ni -O_{CA}	2.050(2)<br>2.081(2)	N_{AA}-C_{α}	1.488(4)<br>1.496(3)
N_{Im}-C_{Im}	1.385(3)<br>1.382(4)	O_{CA}-C_{CA}	1.275(3)<br>1.270(4)	C_{α}-C_{β}	1.564(3)<br>1.562(3)
C_{Im}-C_{ald}	1.505(4)<br>1.503(4)	C_{CA}-C_{α}	1.542(4)<br>1.534(4)	C_{β} -S	1.839(3)<br>1.834(3)
C_{ald}-N_{AA}	1.481(4)<br>1.487(3)	C_{α}-N_{AA}	1.488(4)<br>1.496(3)	S-C_{ald}	1.831(3)<br>1.827(2)
N_{AA}-Ni	2.122(2)<br>2.106(2)	N_{AA}-Ni	2.122(2)<br>2.106(2)	C_{ald} -N_{AA}	1.481(4)<br>1.487(3)
Ni- N_{Im} -C_{Im}	115.40(18)<br>112.93(18)	Ni -O_{CA}-C_{CA}	117.54(17)<br>114.60(19)	N_{AA}-C_{α}-C_{β}	111.7(2)<br>110.7(2)
N_{Im}-C_{Im}-C_{ald}	116.8(2)<br>119.1(2)	O_{CA}-C_{CA}-C_{α}	117.7(2)<br>118.5(2)	C_{α} -C_{β} -S	103.29(19)<br>102.76(18)
C_{Im}-C_{ald} -N_{AA}	109.5(2)<br>110.1(2)	C_{CA}-C_{α}-N_{AA}	112.6(2)<br>111.7(2)	C_{β}-S-C_{ald}	91.28(13)<br>91.51(12)
C_{ald}-N_{AA}-Ni	109.63(16)<br>110.48(15)	C_{α}-N_{AA}-Ni	110.19(15)<br>110.37(17)	S-C_{ald} -N_{AA}	105.02(19)<br>105.91(16)
N_{AA}-Ni—N_{Im}	80.22(9)<br>81.34(9)	N_{AA}-Ni-O_{CA}	81.34(8)<br>81.55(9)	C_{ald}-N_{AA}-C_{α}	111.89(19)<br>112.48(19)

Supplemental Table S3 continued

e) $Ni(LHC^{Tn}4Im)_2$. Imidazole: ($Ni\ N_{Im}\ C_{Im}\ C_{ald}\ N_{AA}$), Carboxylate: ($Ni\ O_{CA}\ C_{CA}\ C_{\alpha}\ N_{AA}$) and Thiazine: ($N_{AA}\ C_{\alpha}\ C_{\beta}\ C_{\gamma}\ SC_{ald}$) There are two values for each entry if there are two ligands in the asymmetric unit.

Imidazole	Value	Carboxylate	Value	Thiazine	value
$Ni\text{-}N_{Im}$	2.0498(18)<br>2.0421(18)	$Ni\text{-}O_{CA}$	2.0742(15)<br>2.0408(15)	$N_{AA}\text{-}C_{\alpha}$	1.492(3)<br>1.494(3)
$N_{Im}\text{-}C_{Im}$	1.383(3)<br>1.376(3)	$O_{CA}\text{-}C_{CA}$	1.269(3)<br>1.269(3)	$C_{\alpha}\text{-}C_{\beta}$	1.532(3)<br>1.529(3)
$C_{Im}\text{-}C_{ald}$	1.498(3)<br>1.505(3)	$C_{CA}\text{-}C_{\alpha}$	1.539(3)<br>1.540(3)	$C_{\beta}\text{-}C_{\gamma}$	1.523(3)<br>1.520(3)
$C_{ald}\text{-}N_{AA}$	1.483(3)<br>1.488(3)	$C_{\alpha}\text{-}N_{AA}$	1.491(3)<br>1.494(3)	$C_{\gamma}\text{-}S$	1.814(2)<br>1.814(2)
$N_{AA}\text{-}Ni$	2.1362(18)<br>2.1531(18)	$N_{AA}\text{-}Ni$	2.1362(18)<br>2.1531(18)	$S\text{-}C_{ald}$	1.823(2)<br>1.817(2)
				$C_{ald}\text{-}N_{AA}$	1.483(3)<br>1.488(3)
$Ni\text{-}N_{Im}\text{-}C_{Im}$	110.35(14)<br>111.70(14)	$Ni\text{-}O_{CA}\text{-}C_{CA}$	115.01(13)<br>115.13(13)	$N_{AA}\text{-}C_{\alpha}\text{-}C_{\beta}$	115.58(16)<br>115.58(16)
$N_{Im}\text{-}C_{Im}\text{-}C_{ald}$	117.74(18)<br>117.46(18)	$O_{CA}\text{-}C_{CA}\text{-}C_{\alpha}$	116.99(18)<br>117.14(18)	$C_{\alpha}\text{-}C_{\beta}\text{-}C_{\gamma}$	112.43(18)<br>113.20(17)
$C_{Im}\text{-}C_{ald}\text{-}N_{AA}$	110.43(16)<br>110.10(17)	$C_{CA}\text{-}C_{\alpha}\text{-}N_{AA}$	110.96(16)<br>111.04(15)	$C_{\beta}\text{-}C_{\gamma}\text{-}S$	109.61(16)<br>109.39(16)
$C_{ald}\text{-}N_{AA}\text{-}Ni$	105.23(13)<br>105.23(12)	$C_{\alpha}\text{-}N_{AA}\text{-}Ni$	106.35(12)<br>105.39(12)	$C_{\gamma}\text{-}S\text{-}C_{ald}$	96.40(10)<br>95.97(10)
$N_{AA}\text{-}Ni\text{—}N_{Im}$	83.90(7)<br>83.19(7)	$N_{AA}\text{-}Ni\text{-}O_{CA}$	81.72(6)<br>82.50(6)	$S\text{-}C_{ald}\text{-}N_{AA}$	116.83(14)<br>116.86(14)
				$C_{ald}\text{-}N_{AA}\text{-}C_{\alpha}$	118.23(17)<br>117.52(17)

Supplemental Table S4 Summary of %yield, EA and ESI MS values for one enantiomer of the prepared complexes

Complex	%Yield	Theoretical CHN (%)	Experimental CHN (%)	ESI (m/e)
Ni(LC^{Tz}4Im)$_2$ · 2.5H$_2$O	76%	33.62<br>4.23<br>16.8	33.72<br>3.99<br>16.67	[MNa]$^+$ 477
Ni(LC^{Tz}2Me4Im)$_2$ · H$_2$O	68%	38.34<br>4.42<br>16.77	38.34<br>4.18<br>16.49	[MH]$^+$ 483<br>[MNa]$^+$ 505<br>[MK]$^+$ 521<br>[Ni$_2$L$_3$]$^+$ 752
Ni(LC^{Tz}5Me4Im)$_2$ · H$_2$O	56%	38.34<br>4.42<br>16.77	38.3<br>3.94<br>16.2	[MH]$^+$ 483<br>[MNa]$^+$ 505<br>[MK]$^+$ 521<br>[Ni$_2$L$_3$]$^+$ 752
Ni(LPN^{Tz}5Me4Im)$_2$ · 2H$_2$O · MeOH	53%	41.53<br>5.97<br>13.84	41.39<br>5.56<br>13.70	[MH]$^+$ 539<br>[MNa]$^+$ 561<br>[Ni$_2$L$_3$]$^+$ 836
Ni(LHC^{Tn}4Im)$_2$ · 4.5H$_2$O	63%	34.06<br>5.18<br>14.89	33.85<br>4.82<br>14.53	[MH]$^+$ 483<br>[MNa]$^+$ 505<br>[MK]$^+$ 521
Ni(LγOHI^{Ald}5Me4Im)$_2$ · 4.5H$_2$O	69%	42.88<br>6.71<br>13.64	42.85<br>6.56<br>13.58	[MH]$^+$ 535<br>[Ni$_2$L$_3$]$^+$ 830

molecules

MDPI

Chemical Compounds Characterization Checklist

Manuscript ID: 4375520

Submitting Author: Greg Brewer

Compound				Characterization										
Compound Nr.	New	Known (Ref. in main text)	Physical State (purity %)	1H NMR	13C NMR	2D NMR	HRMS	IR	XRD	GC/ HPLC	Elemental Analysis	+ion ESI MS		Data in SI
2427242	☑		s	☐	☐	☐	☐	☑	☑	☐	☑	☑	☐	☑
2488357	☑		s	☐	☐	☐	☐	☑	☑	☐	☐	☐	☐	☑
2427221	☑		s	☐	☐	☐	☐	☑	☑	☐	☑	☑	☐	☑
2488361	☑		s	☐	☐	☐	☐	☑	☑	☐	☐	☐	☐	☑
2496227	☑		s	☐	☐	☐	☐	☑	☑	☐	☑	☑	☐	☑
2488359	☑		s	☐	☐	☐	☐	☑	☑	☐	☐	☐	☐	☑
2427220	☑		s	☐	☐	☐	☐	☑	☑	☐	☑	☑	☐	☑
2488358	☑		s	☐	☐	☐	☐	☑	☑	☐	☐	☐	☐	☑
2427234	☑		s	☐	☐	☐	☐	☑	☑	☐	☑	☑	☐	☑
2496228	☑		s	☐	☐	☐	☐	☑	☑	☐	☐	☐	☐	☑
2427235	☑		s	☐	☐	☐	☐	☑	☑	☐	☑	☑	☐	☑
	☐			☐	☐	☐	☐	☐	☐	☐	☐	☐	☐	☐

Note: insert the relevant information and select only the techniques used in this study. In the empty columns you can insert any additional methods.

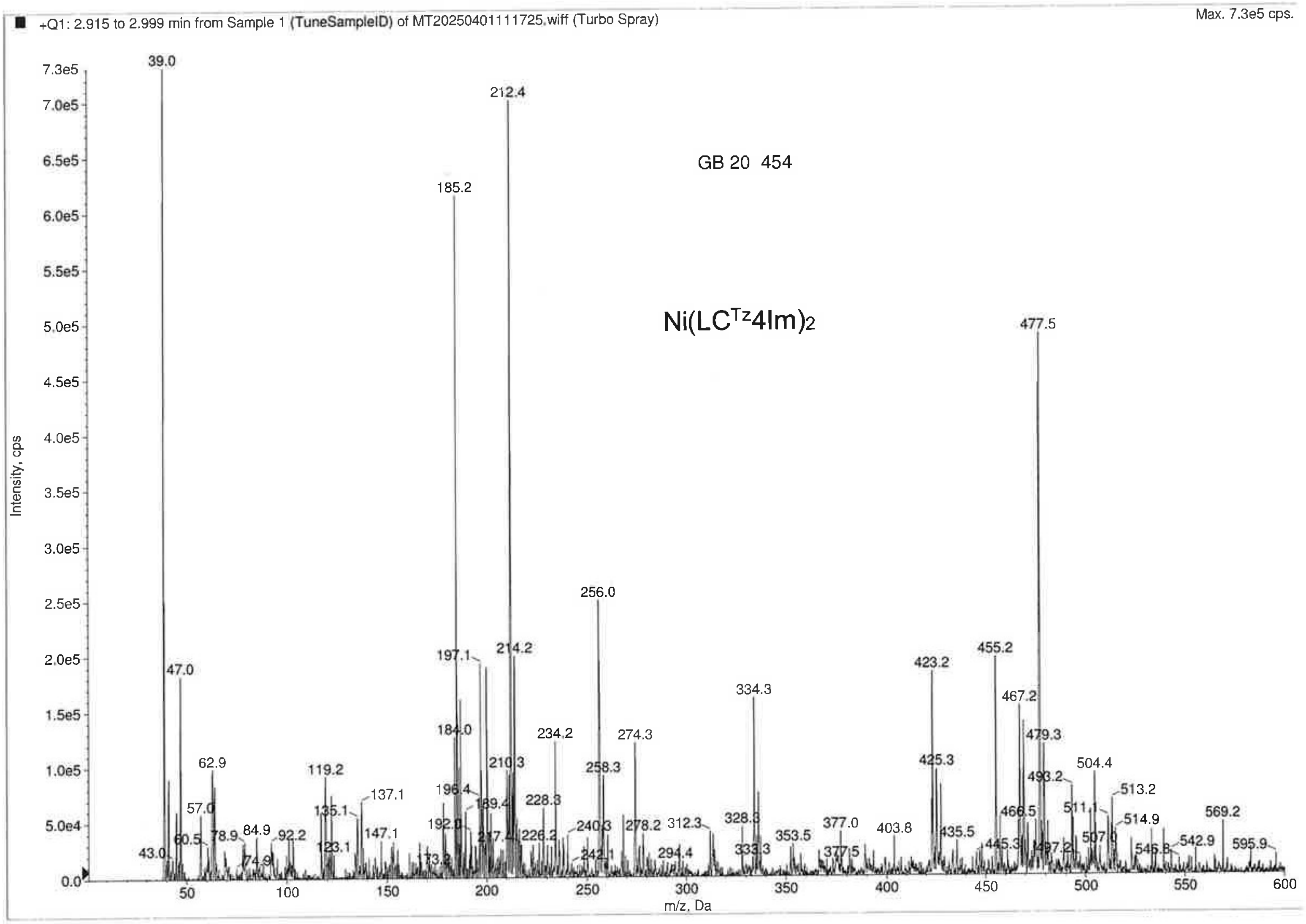
+Q1: 2.915 to 2.999 min from Sample 1 (TuneSampleID) of MT20250401111725.wiff (Turbo Spray)
Max. 7.3e5 cps.
GB 20 454
Ni(LC^{Tz}4Im)$_2$
Intensity, cps
m/z, Da
39.0
212.4
185.2
477.5
256.0
214.2
197.1
455.2
423.2
47.0
334.3
467.2
184.0
234.2
274.3
479.3
62.9
119.2
425.3
504.4
210.3
258.3
137.1
196.4
493.2
513.2
228.3
569.2
57.0
135.1
189.4
466.5
511.1
514.9
377.0
403.8
312.3
328.3
353.5
435.5
542.9
595.9

+Q1: 0.988 to 1.089 min from Sample 1 (TuneSampleID) of MT20251028165833.wiff (Turbo Spray)

Max. 5.8e6

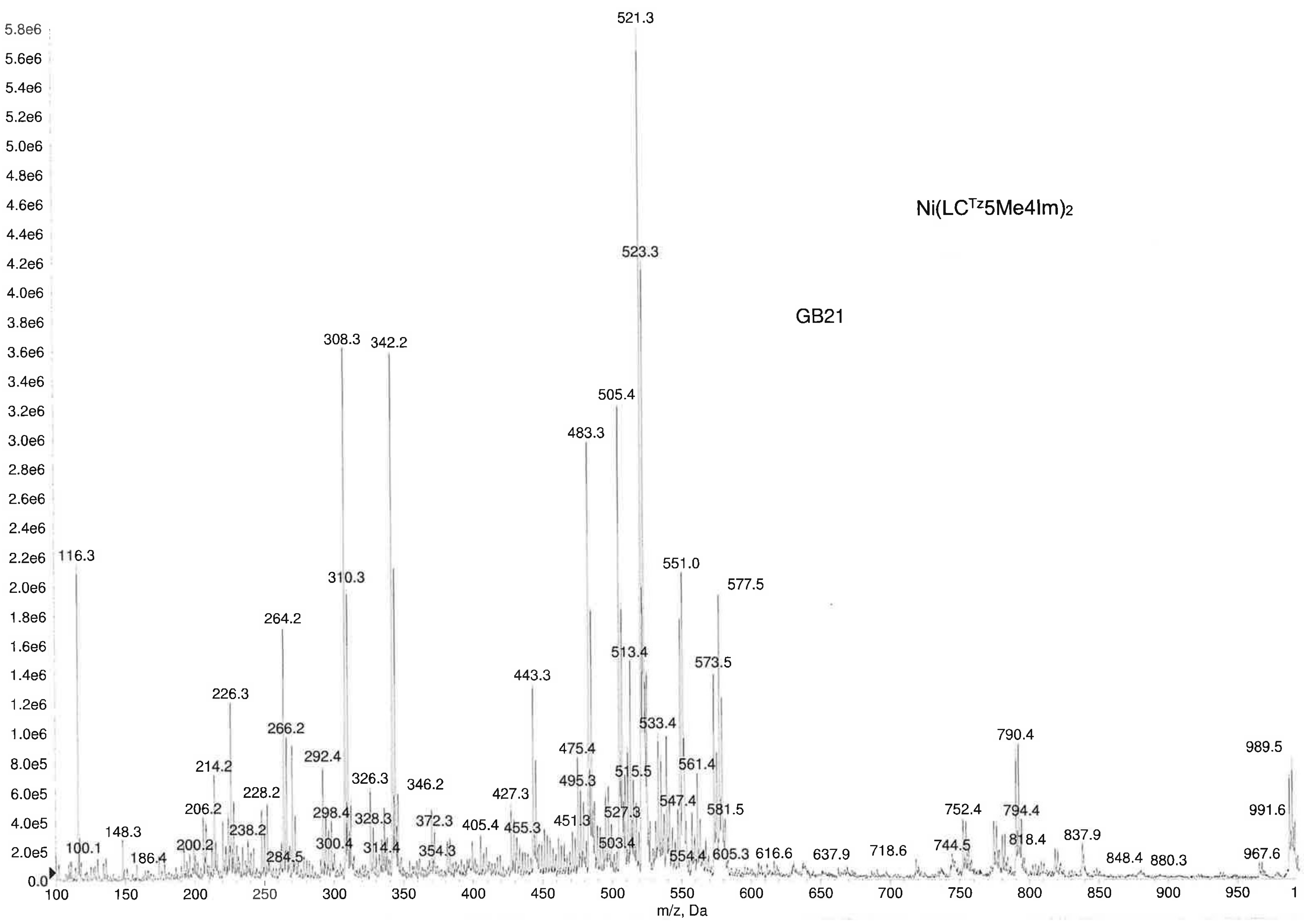

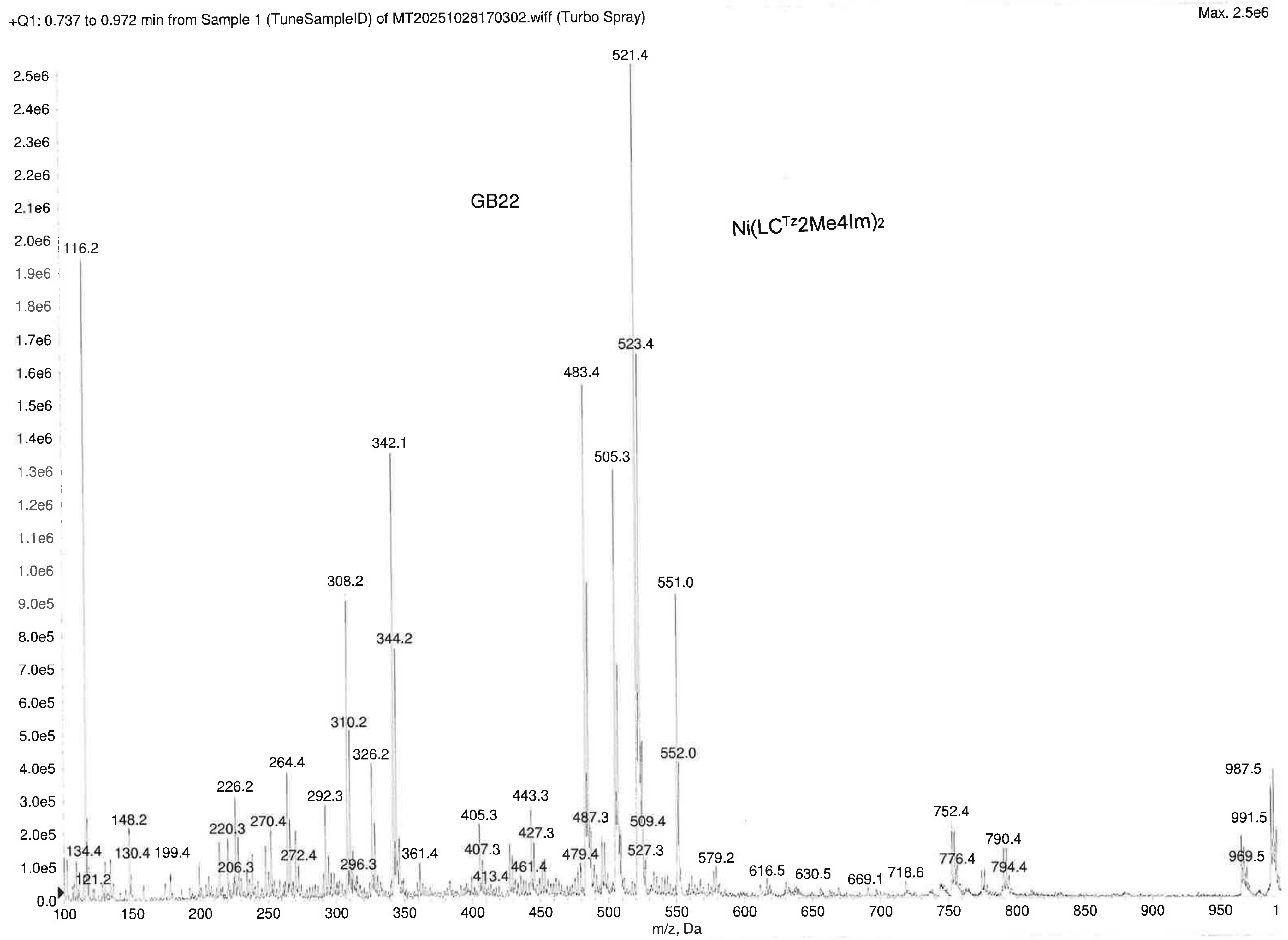
+Q1: 0.737 to 0.972 min from Sample 1 (TuneSampleID) of MT20251028170302.wiff (Turbo Spray)
Max. 2.5e6
GB22
Ni(LC^{Tz}2Me4Im)$_2$
116.2
134.4
121.2
148.2
130.4
199.4
226.2
220.3
206.3
270.4
264.4
272.4
292.3
308.2
310.2
296.3
326.2
342.1
344.2
361.4
405.3
407.3
413.4
443.3
427.3
461.4
483.4
487.3
479.4
505.3
521.4
523.4
509.4
527.3
551.0
552.0
579.2
616.5
630.5
669.1
718.6
752.4
776.4
790.4
794.4
987.5
991.5
969.5
m/z, Da

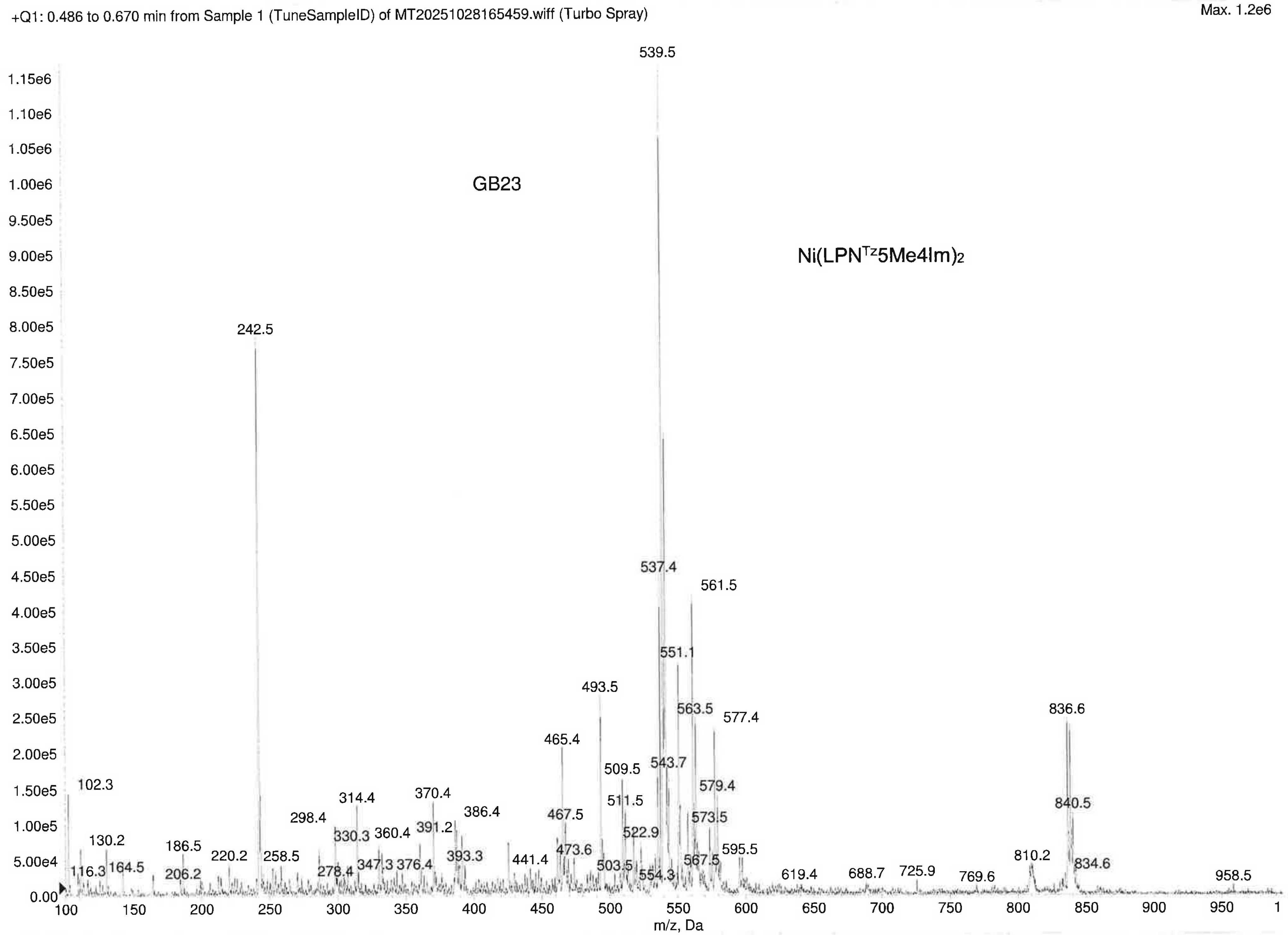
+Q1: 0.486 to 0.670 min from Sample 1 (TuneSampleID) of MT20251028165459.wiff (Turbo Spray)
Max. 1.2e6
GB23
Ni(LPN^{Tz}5Me4Im)$_2$
539.5
242.5
537.4
561.5
551.1
493.5
563.5
577.4
836.6
465.4
509.5
543.7
579.4
102.3
314.4
370.4
298.4
386.4
467.5
511.5
840.5
573.5
330.3
360.4
391.2
522.9
130.2
186.5
220.2
258.5
393.3
473.6
595.5
441.4
810.2
725.9
116.3
164.5
206.2
278.4
347.3
376.4
503.5
554.3
567.5
834.6
619.4
688.7
769.6
958.5
m/z, Da

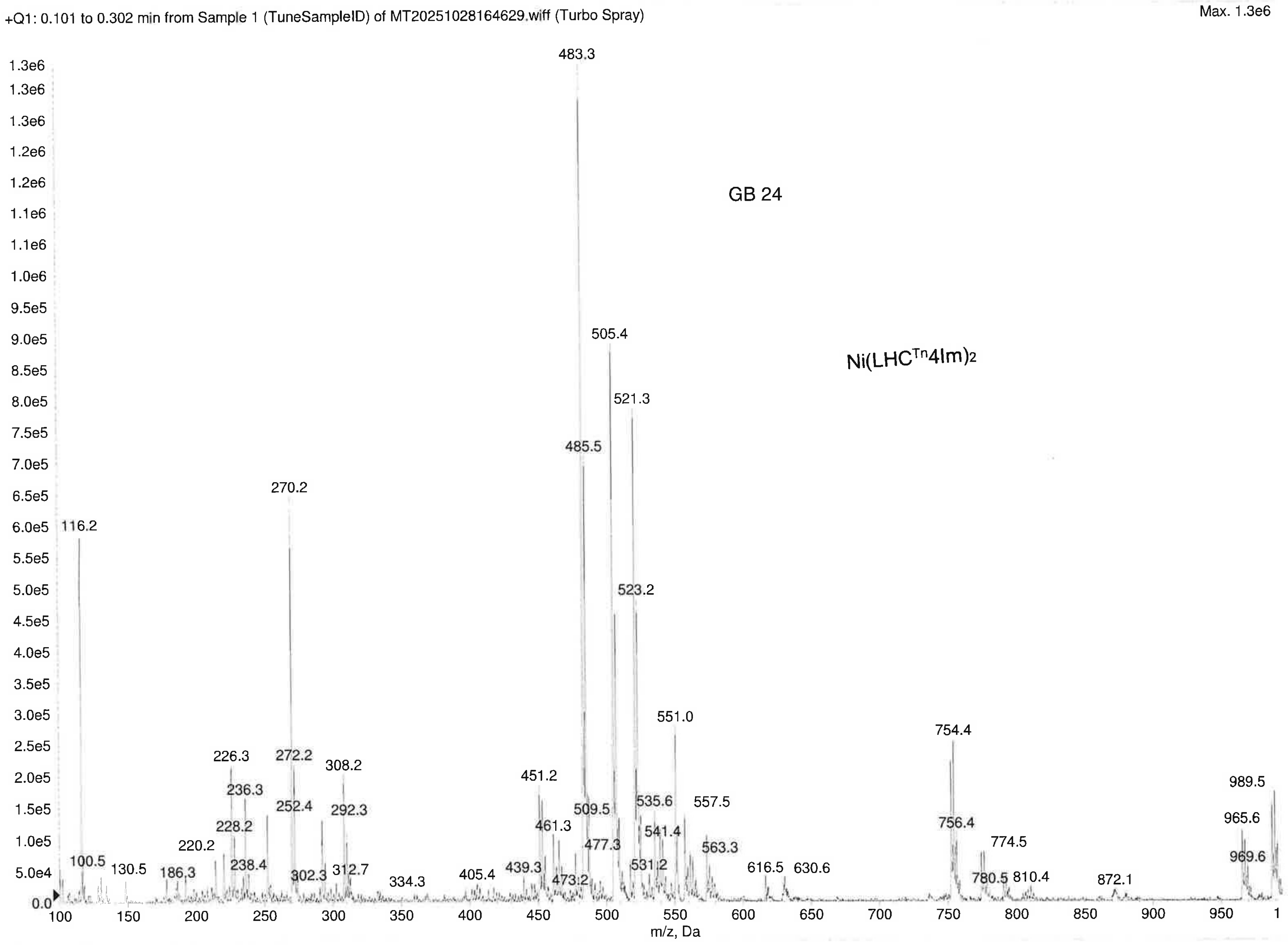

+Q1: 0.101 to 0.302 min from Sample 1 (TuneSampleID) of MT20251028164629.wiff (Turbo Spray)
Max. 1.3e6
GB 24
Ni(LHC^Tn4Im)2
m/z, Da
483.3
505.4
521.3
485.5
270.2
116.2
523.2
551.0
754.4
989.5
965.6
969.6
226.3
272.2
308.2
236.3
252.4
292.3
451.2
509.5
535.6
557.5
756.4
774.5

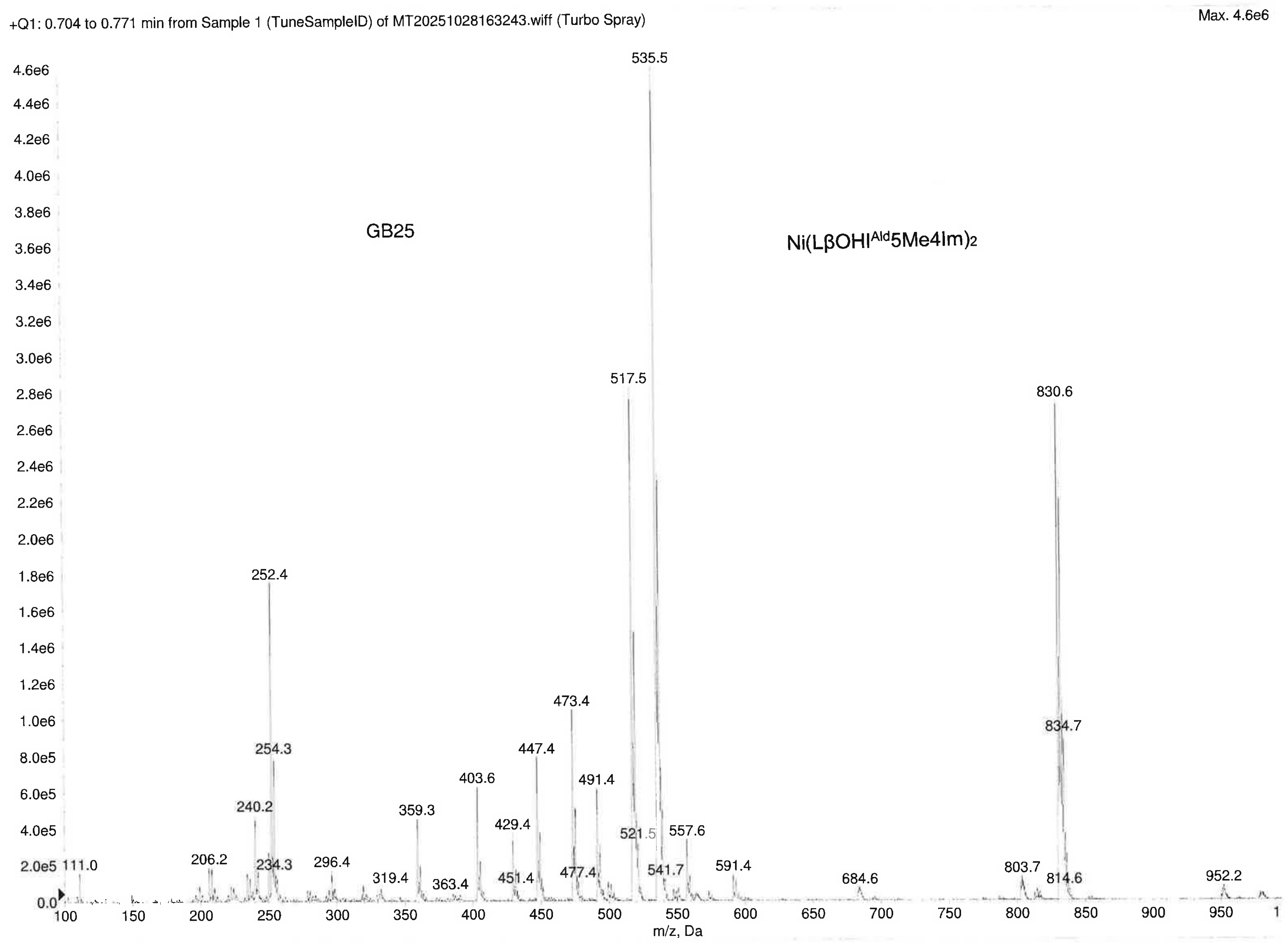
+Q1: 0.704 to 0.771 min from Sample 1 (TuneSampleID) of MT20251028163243.wiff (Turbo Spray)
Max. 4.6e6
GB25
Ni(LβOHI^Ald5Me4Im)2
535.5
517.5
830.6
252.4
473.4
834.7
254.3
447.4
403.6
491.4
240.2
359.3
429.4
557.6
521.5
111.0
206.2
234.3
296.4
591.4
319.4
363.4
451.4
477.4
541.7
684.6
803.7
814.6
952.2
4.6e6
4.4e6
4.2e6
4.0e6
3.8e6
3.6e6
3.4e6
3.2e6
3.0e6
2.8e6
2.6e6
2.4e6
2.2e6
2.0e6
1.8e6
1.6e6
1.4e6
1.2e6
1.0e6
8.0e5
6.0e5
4.0e5
2.0e5
0.0
100
150
200
250
300
350
400
450
500
550
600
650
700
750
800
850
900
950
1
m/z, Da

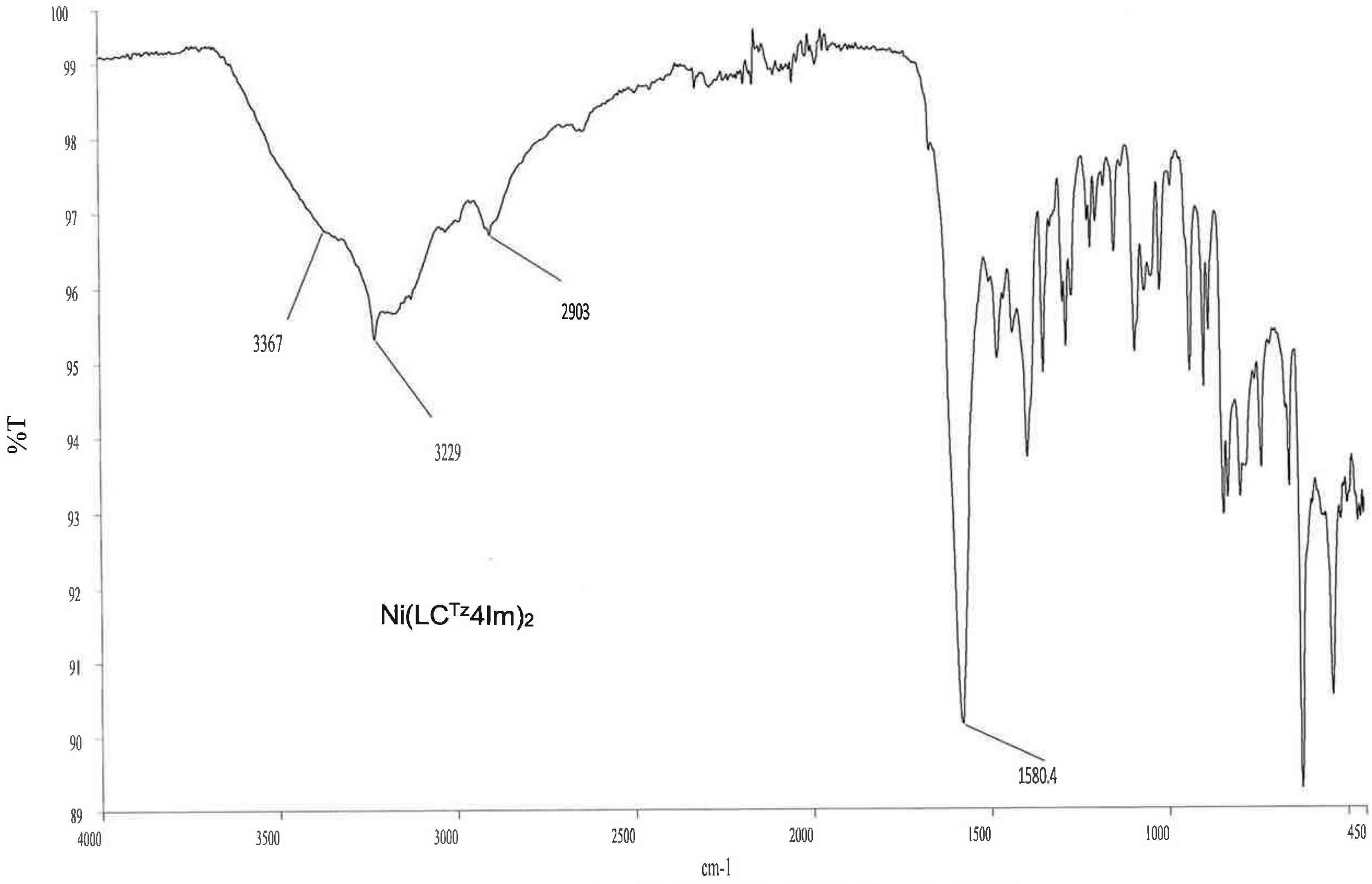

Sample 116 By Administrator Date Monday, September 15 2025

J70 - 1169

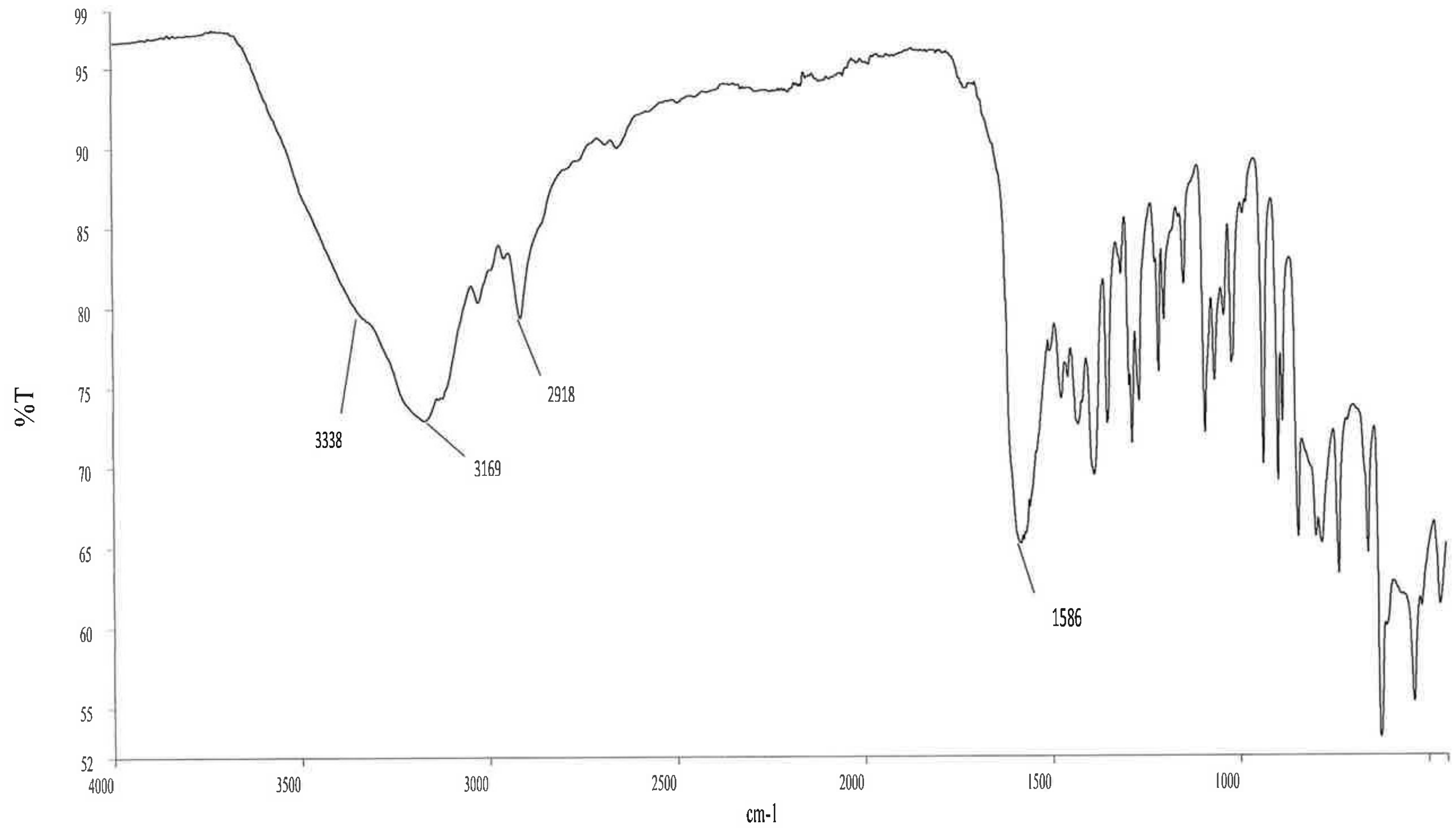

K77-1

$Ni(DC^{Tz}4Im)_2$

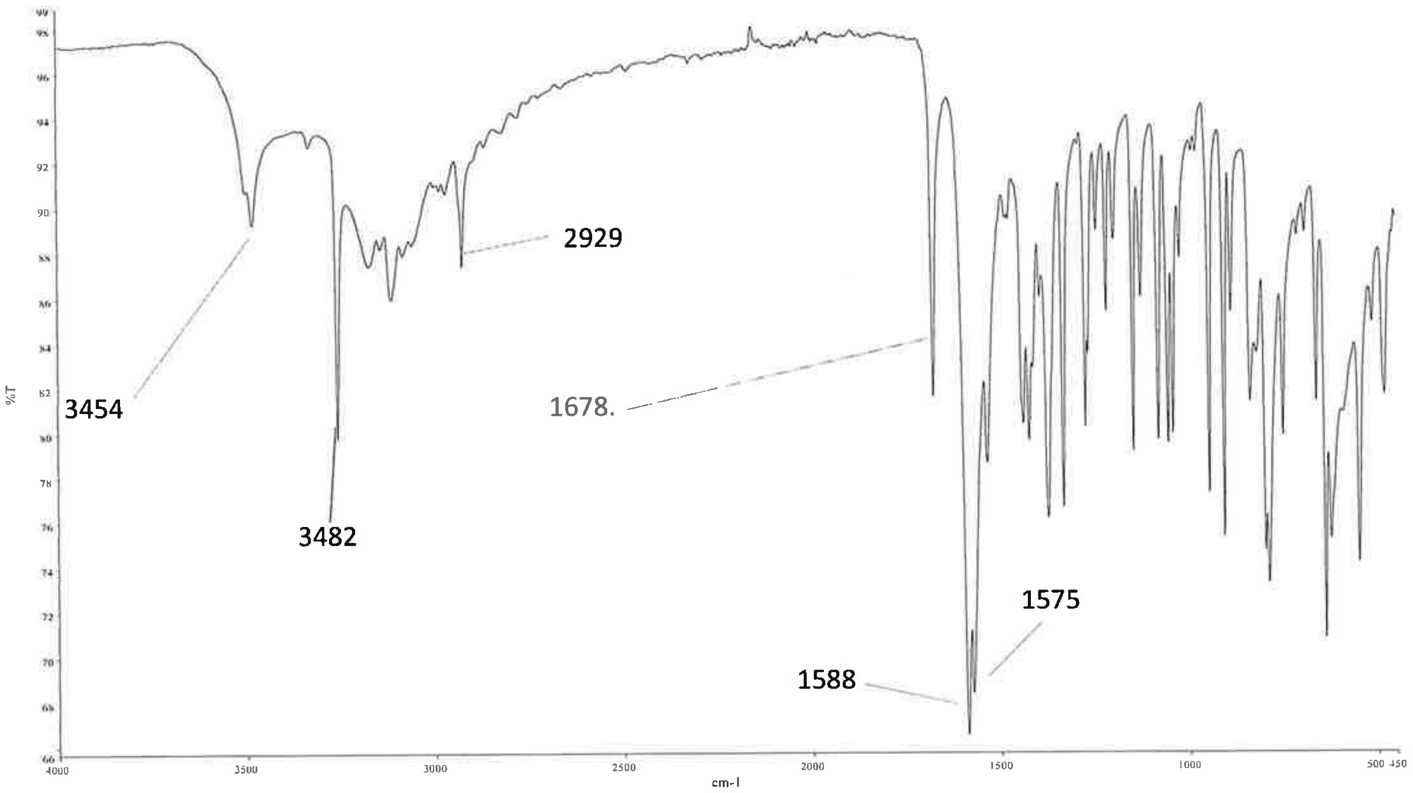

K65-1

Ni(LC^{Tz}2Me4Im)$_2$

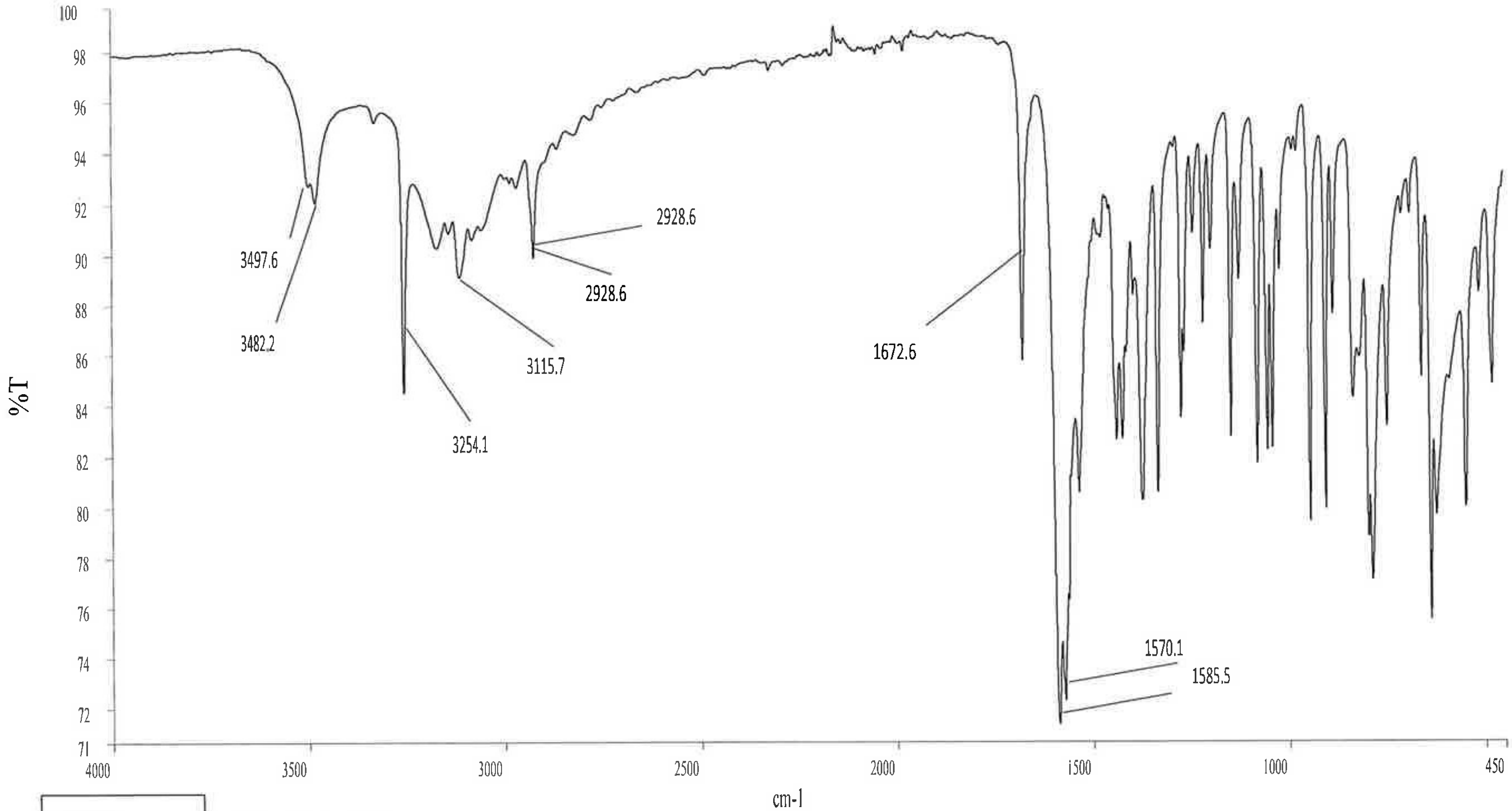

K77-2

Administrator 1170

Ni(DC^{Tz}2Me4Im)$_2$

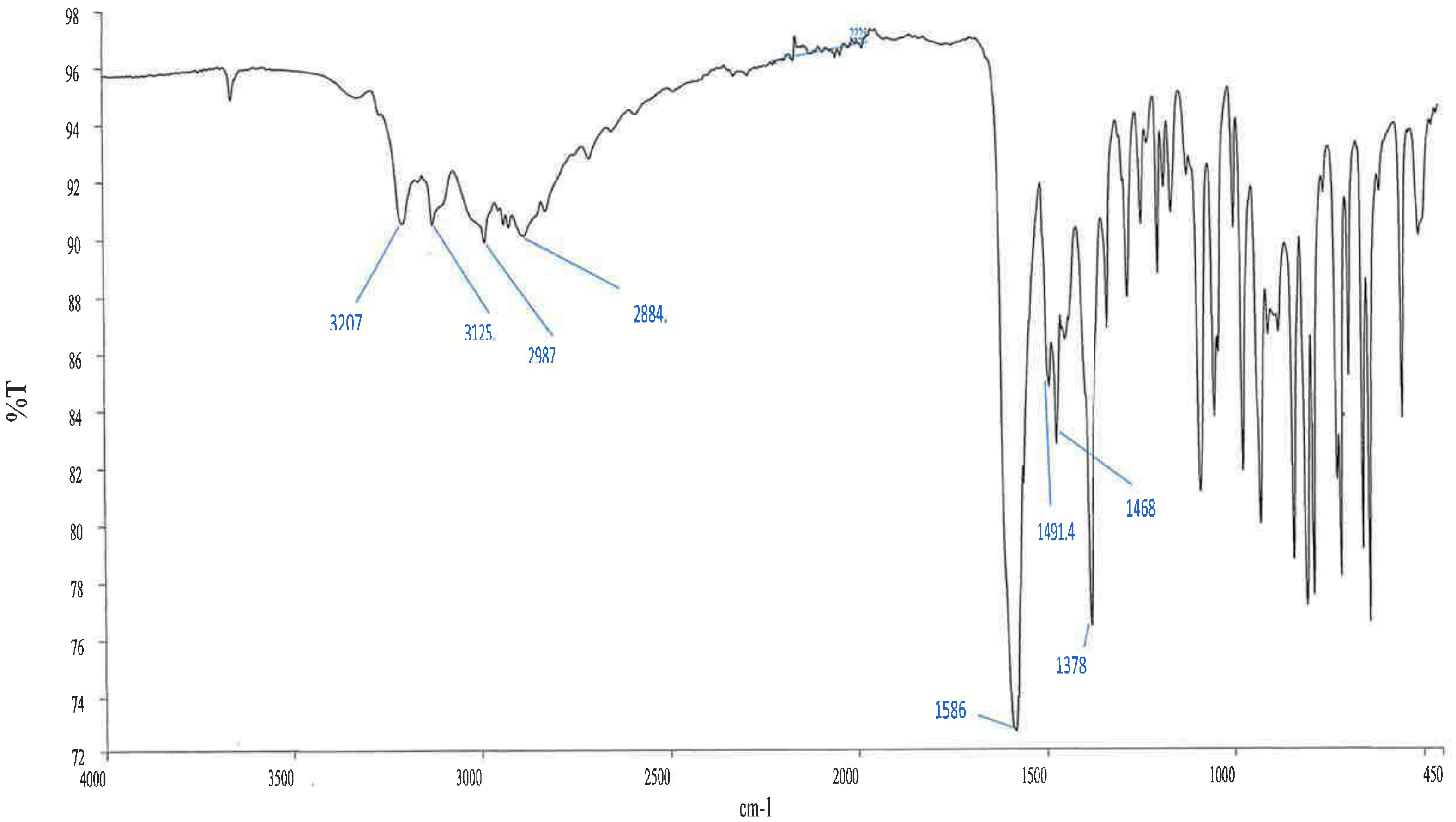

K93

Ni(LC^{Tz}5Me4Im)$_2$

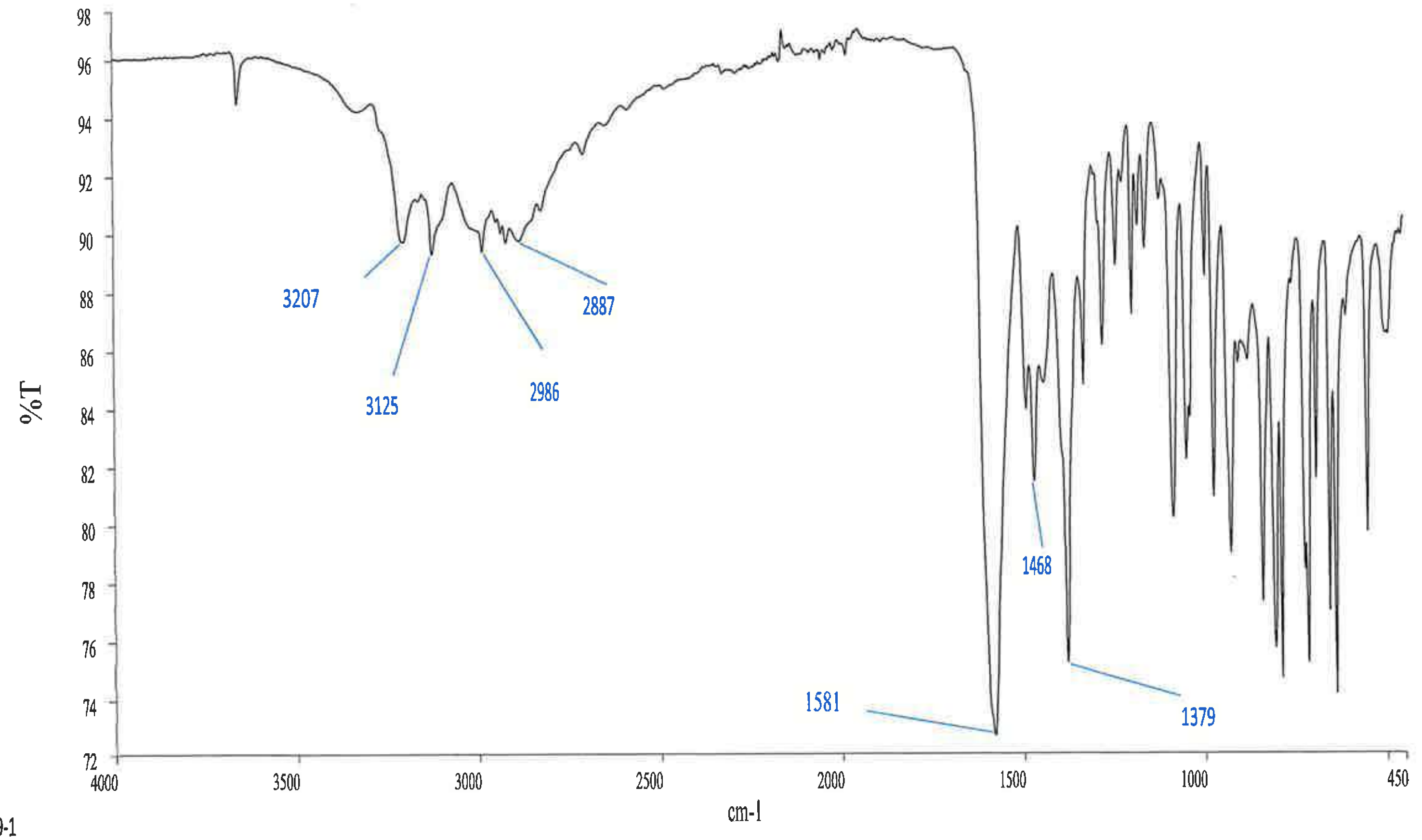

K89-1

Ni(DC^{Tz}5Me4Im)$_2$

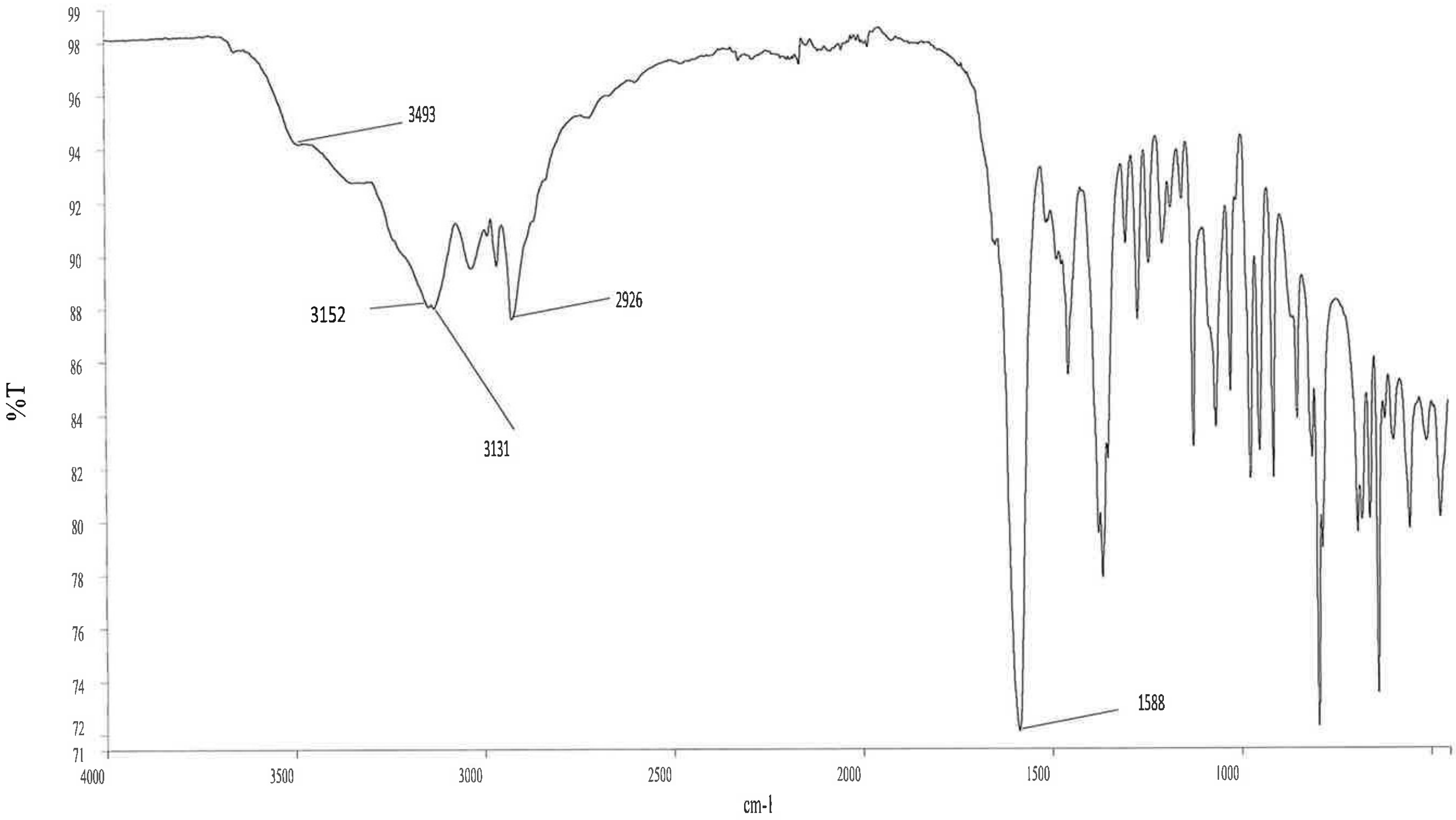

K64-2

$Ni(LPN^{Tz}5Me4Im)_2$

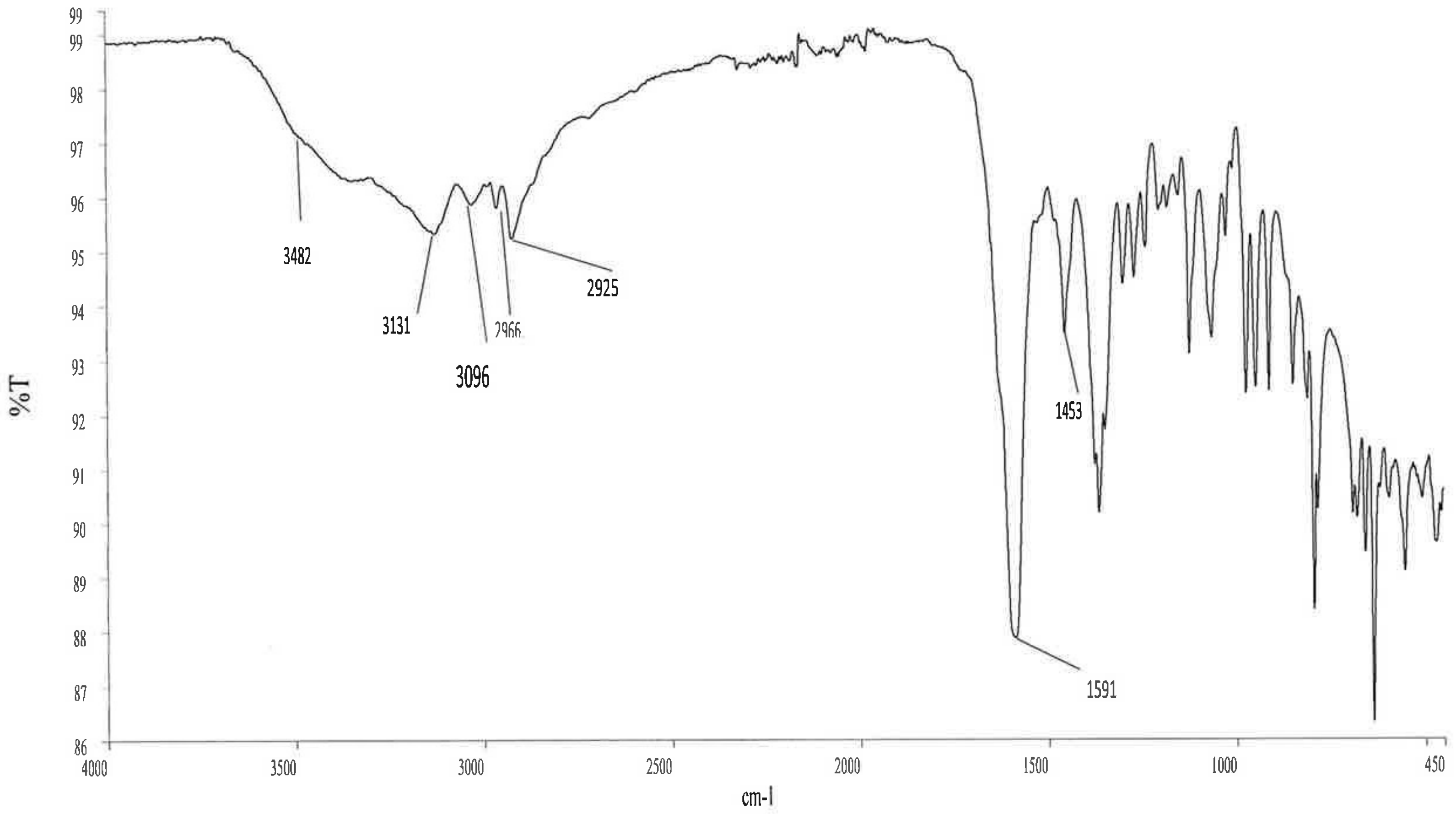

Ni(DPN^{Tz}5Me4Im)$_2$

K84-1

Administrato

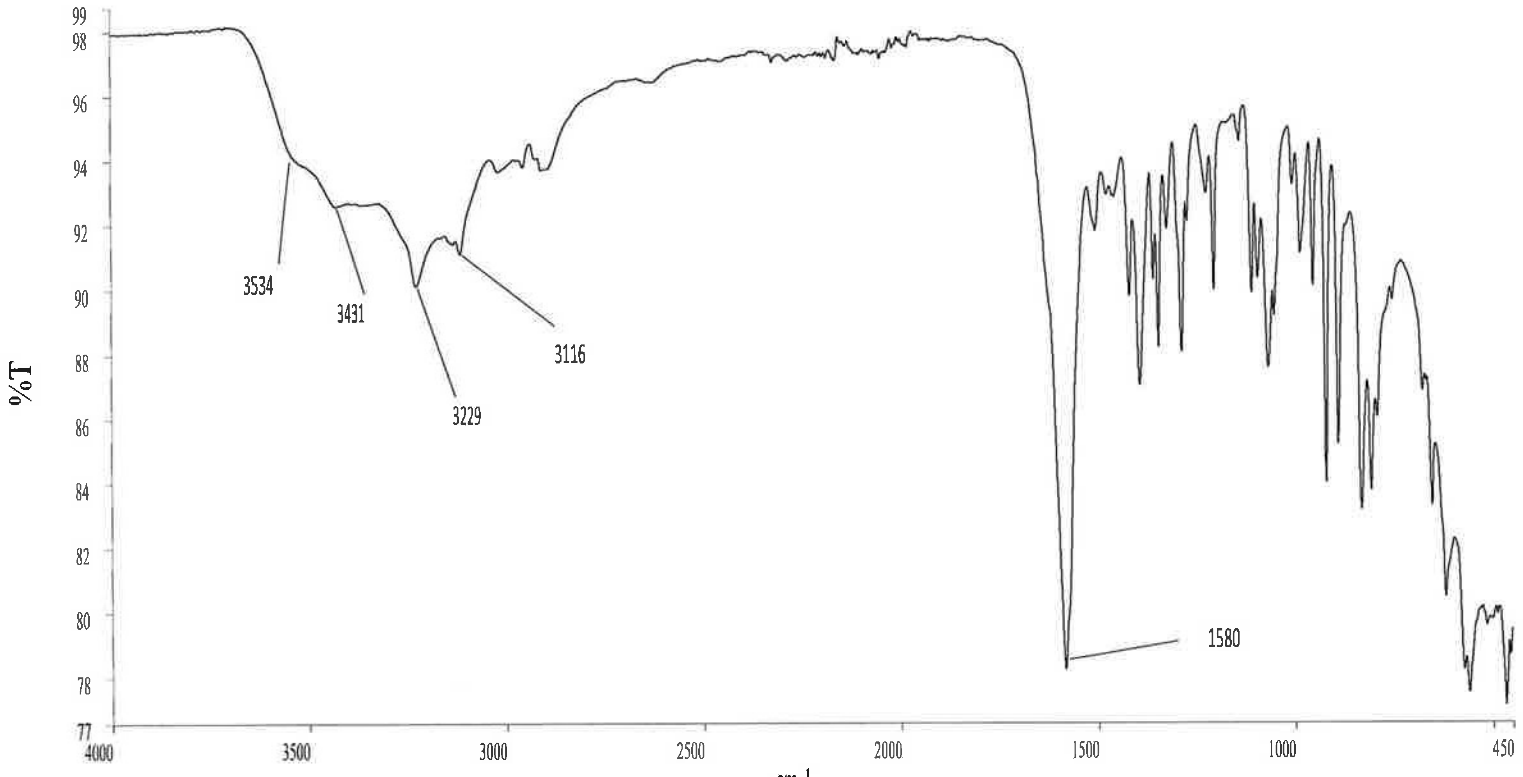

K 49-1 (1168)

$Ni(LHC^{Tn}4Im)_2$

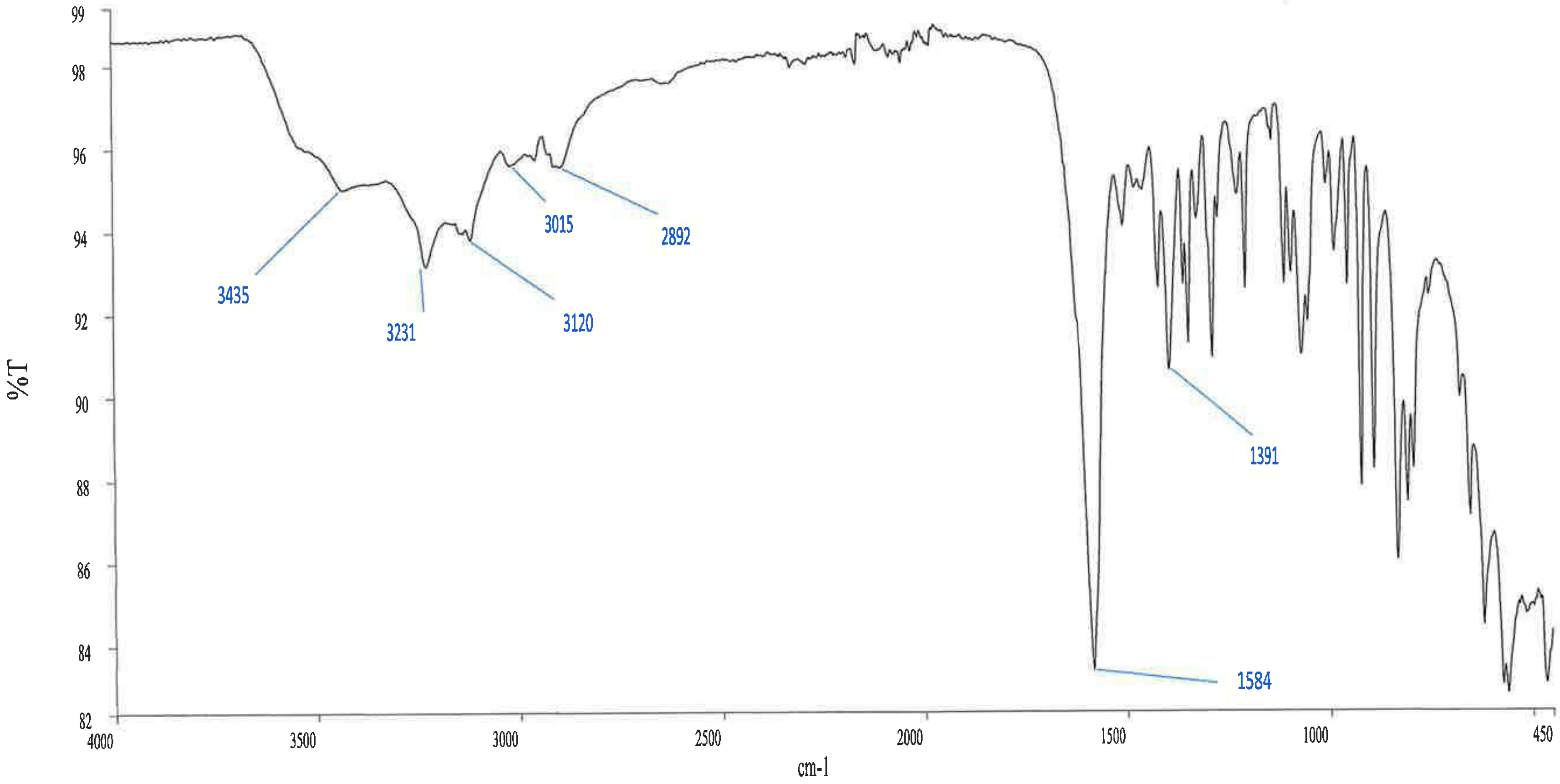

K94

Ni(DHC^{Tn}4Im)$_2$

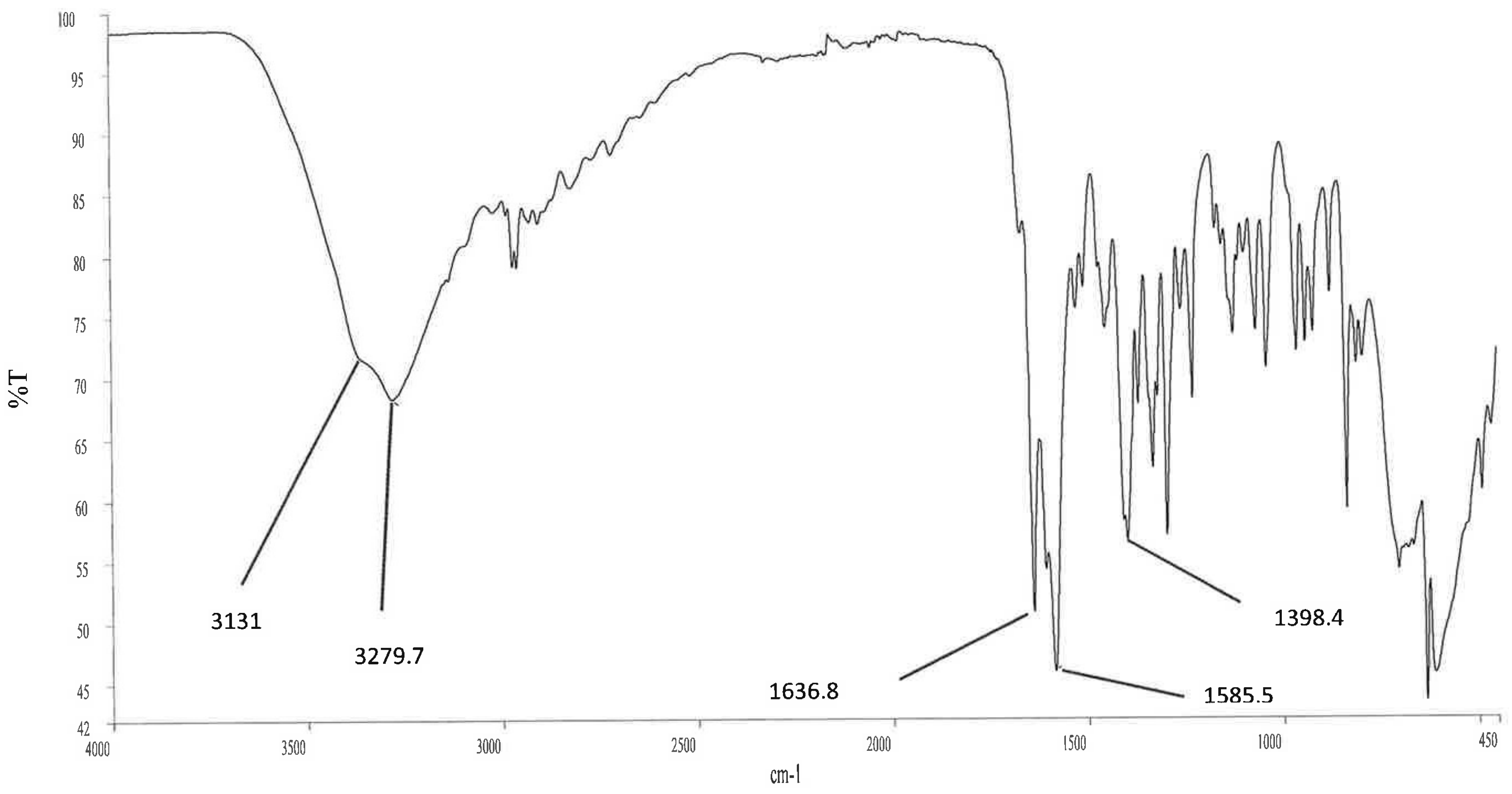

1167 K37-2

Ni(LβOHI^{Ald}5Me4Im)$_2$
